# Recent Advances in the Understanding of Specific Efferent Pathways Emerging From the Cerebellum

**DOI:** 10.3389/fnana.2021.759948

**Published:** 2021-12-16

**Authors:** Seulgi Kang, Soyoung Jun, Soo Ji Baek, Heeyoun Park, Yukio Yamamoto, Keiko Tanaka-Yamamoto

**Affiliations:** ^1^Brain Science Institute, Korea Institute of Science and Technology (KIST), Seoul, South Korea; ^2^Division of Bio-Medical Science and Technology, KIST School, University of Science and Technology (UST), Seoul, South Korea

**Keywords:** cerebellum, viral tracers, optogenetics, chemogenetics, higher cognitive functions, neuronal networks, deep cerebellar nuclei (DCN)

## Abstract

The cerebellum has a long history in terms of research on its network structures and motor functions, yet our understanding of them has further advanced in recent years owing to technical developments, such as viral tracers, optogenetic and chemogenetic manipulation, and single cell gene expression analyses. Specifically, it is now widely accepted that the cerebellum is also involved in non-motor functions, such as cognitive and psychological functions, mainly from studies that have clarified neuronal pathways from the cerebellum to other brain regions that are relevant to these functions. The techniques to manipulate specific neuronal pathways were effectively utilized to demonstrate the involvement of the cerebellum and its pathways in specific brain functions, without altering motor activity. In particular, the cerebellar efferent pathways that have recently gained attention are not only monosynaptic connections to other brain regions, including the periaqueductal gray and ventral tegmental area, but also polysynaptic connections to other brain regions, including the non-primary motor cortex and hippocampus. Besides these efferent pathways associated with non-motor functions, recent studies using sophisticated experimental techniques further characterized the historically studied efferent pathways that are primarily associated with motor functions. Nevertheless, to our knowledge, there are no articles that comprehensively describe various cerebellar efferent pathways, although there are many interesting review articles focusing on specific functions or pathways. Here, we summarize the recent findings on neuronal networks projecting from the cerebellum to several brain regions. We also introduce various techniques that have enabled us to advance our understanding of the cerebellar efferent pathways, and further discuss possible directions for future research regarding these efferent pathways and their functions.

## Introduction

The cerebellum is a region of the brain that is anatomically separated from the other regions, and is morphologically unique in several aspects. The foliated structure of the cerebellum is composed of three well-defined layers, i.e., the granular layer, Purkinje cell layer, and molecular layer, with white matter in the center of each lobule (Eccles et al., 1967). There are only a few major types of neurons in the cerebellar cortex, and they are strictly located in their designated layers. One of the two major cerebellar inputs comes from mossy fibers, which originate from several regions of the brainstem and spinal cord, and indirectly innervates Purkinje cells (PCs) through granule cells. Another type of major inputs, from climbing fibers, directly innervates PCs. PCs are inhibitory output neurons from the cerebellar cortex, and their axons mainly project to the deep cerebellar nuclei (DCN), from where cerebellar efferent projections are sent to other brain regions.

Whereas the cerebellum has traditionally been considered to be important solely for motor coordination and learning, it became apparent that it is also involved in non-motor functions, such as cognitive and psychological functions (Rochefort et al., 2013; Phillips et al., 2015; Moreno-Rius, 2019; Hull, 2020; Wagner and Luo, 2020). Because the basic network architectures are uniform throughout the cerebellum (Cerminara et al., 2015; Beckinghausen and Sillitoe, 2019), the cerebellum is thought to utilize the same network structures for various brain functions, via segregated regions within the cerebellum that receive different types of mossy fiber or climbing fiber inputs and send neuronal signals to different brain regions. Indeed, beyond the traditional cerebellar functions in supervised motor learning utilizing prediction error, lines of evidence suggest that the cerebellar network machinery is widely involved in the processing, generating, and testing of motor and non-motor predictions (Sokolov et al., 2017; Popa and Ebner, 2018; Hull, 2020). Furthermore, it has been shown that the cerebellum makes broad projections to multiple brain regions through both direct and indirect pathways (Kebschull et al., 2020). Thus, specific anatomical efferent connections appear to at least be partly responsible for specific cerebellar functions.

The DCN, which is a source of cerebellar outputs, is basically composed of three nuclei, namely, the dentate (DN), interpositus (IPN), and fastigial (FN) nuclei (Sugihara, 2011; Ruigrok and Teune, 2014; Thanawalla et al., 2020), and the IPN can be further subdivided to two regions, anterior and posterior IPN. Owing to the zonal organization between the cerebellar cortex and DCN (Houck and Person, 2015), the DN, IPN, and FN mainly receive synaptic inputs from PCs located in the hemisphere, pars intermedia (paravermis), and vermis of the ipsilateral cerebellar cortex, respectively. Whereas PCs send gamma-aminobutyric acid-ergic (GABAergic) inhibitory inputs to DCN neurons, collaterals of mossy fibers and climbing fibers send glutamatergic excitatory inputs to them (Sugihara, 2011; Thanawalla et al., 2020). All three nuclei of the DCN are embedded in the deep central area of the cerebellum. Even though DCN neurons in general project to a broad area, the overall projecting targets from each nucleus have different characteristics (Teune et al., 2000; Kebschull et al., 2020), such as more projections to the brainstem from the FN and more projections to the thalamus or midbrain from the IPN or DN. The anterior and posterior IPNs were also shown to have different projecting patterns (Teune et al., 2000; Lu et al., 2012). This suggests that each nucleus contributes to distinct functions. Detailed analyses further demonstrated different projection patterns from distinct groups of neurons in the same nucleus (Fujita et al., 2020; Kebschull et al., 2020). Heterogeneity in the electrophysiological and anatomical properties has also been reported in DCN neurons (Czubayko et al., 2001; Aizenman et al., 2003; Sultan et al., 2003; Uusisaari and Knöpfel, 2012; Canto et al., 2016), although their functional relevance is not completely understood. A part of the heterogeneity arises from the three different types of projecting neurons (Baumel et al., 2009; Thanawalla et al., 2020). The majority of projecting neurons are glutamatergic neurons, which project to most of the extracerebellar target regions and send feedback signals to the cerebellar cortex (Houck and Person, 2015; Gao et al., 2016). Other well-known projecting neurons are GABAergic neurons, which specifically send feedback projections to the origin of the climbing fibers, i.e., the inferior olive (IO) (De Zeeuw et al., 1998), although a very recent study observed broad projections of GABAergic DCN neurons (Judd et al., 2021). A small population of glycinergic projecting neurons are also found in the FN (Bagnall et al., 2009). In addition to the outputs from the DCN, particular populations of PCs also directly project to specific nuclei in the brainstem (Sekirnjak et al., 2003; Schwarz et al., 2015; Hashimoto et al., 2018). Thus, unlike relatively uniform network structures in the cerebellar cortex, efferent projections mostly from DCN neurons and occasionally from PCs are highly heterogeneous, and the investigation of efferent pathways is essential toward understanding the multifunctionality of the cerebellum. Fortunately, studies on cerebellar efferent pathways have been actively conducted in recent years. In this review article, we introduce information obtained from these studies mainly in rodents using cutting-edge techniques. We first briefly touch on the long-known cerebellar efferent pathways and their motor functions, and then describe recent evidence on efferent pathways to other brain regions from the aspect of non-motor cerebellar functions. Behavioral functions of efferent pathways demonstrated in recent studies are summarized in Table 1, although further studies are necessary to test possibilities that these individual pathways are also involved in other functions. We acknowledge the possibility that the compartmental organization of the DCN is relevant to functions of the efferent pathways, because this organization is closely associated with the striped modular organization of the cerebellar cortex that is based on the expression of zebrin II (also known as aldorase C) in PCs (Sugihara, 2011). Various studies have shown the functional and structural relevance of the striped modular organization of the cerebellar cortex (De Zeeuw and Ten Brinke, 2015; Tsutsumi et al., 2015; Zhou J. et al., 2020; Beekhof et al., 2021). However, we will not consider the compartmental organization of the DCN in this article, because it is not yet clear how this organization is associated with the historically less well studied efferent pathways involved in non-motor cerebellar functions, which are the main topics of this article.

**TABLE 1:** Behavioral analyses demonstrating non-primary motor functions of cerebellar efferent pathways that have been recently demonstrated (gray and pink).

Category		Cerebellar efferent pathways	Functions	Behavior test (species used)	References
	
Historically studied pathways	Motor	Flocculus – vestibular nucleus	Motor learning	VOR (mouse)	Jang et al., 2020
	functions	Anterior IPN – mRN	Associated motor learning	Delay eyeblink conditioning	Freeman and Steinmetz, 2011 (review)
		Vermis – FN – ventral medullary reticular formation	Associated motor learning	Delay eyeblink conditioning (mouse)	Wang et al., 2020
		IPN/DN – ventral anterolateral thalamus – motor cortex (caudal forelimb area)	Context-dependent movement initiation	Cued forelimb push task (mouse)	Dacre et al., 2021
		Lateral Crus I – motor thalamus -primary sensory/motor cortex	Control of voluntary movements	Whisker tracking (mouse)	Proville et al., 2014
		Anterior IPN – ventral anterior, ventral lateral thalamus or mRN	Modulation of limb movements	Skilled reaching task Gait analyses of freely moving mice (mouse)	Low et al., 2018
		Anterior IPN – ipsilateral cervical spinal cord	Skilled forelimb performance	Single pellet reaching task (mouse)	Sathyamurthy et al., 2020
		FN/posterior IPN – contralateral cervical spinal cord	Skilled locomotor learning	Accelerating rotarod task (mouse)	
		Anterior IPN – IO	Extinction of associated motor learning	Eyeblink conditioning (mouse)	Kim et al., 2020
		FN – IO	State changes underlying skilled movement	Targeted arm-reaching task (mouse)	Wagner et al., 2021
Recently demonstrated pathways		FN – ventral anterior lateral thalamic nucleus – ALM	Motor planning	Sensory discrimination task and learned directional movement (mouse)	Gao et al., 2018
		DN – ALM	Preparatory behavior prior to goal-directed movement	Virtual reality conditioning task (mouse)	Chabrol et al., 2019
	Non-motor	DCN – VTA functions	Reward Social behavior	Conditioned place preference test (mouse) Three-chamber social behavior task (mouse)	Carta et al., 2019
		Crus I – DN, IPN – dorsolateral posterior VTA	Development of depressive symptoms	Tail suspension test, forced swim test, novelty-suppressed feeding test, sucrose splash test (mouse)	Baek et al., 2021
		FN – vlPAG	Control of fear memory	Fear conditioning (mouse)	Frontera et al., 2020
		FN – vlPAG – magnocellular reticular nucleus	Freezing behavior	Motion detection (mouse)	Vaaga et al., 2020
		Anterior vermis – FN – DRN	Antidepressant action	Forced swim test, novelty-suppressed feeding test, sucrose preference test (rat)	Bambico et al., 2018
		Crus I – DN – ventromedial thalamus– mPFC	Regulation of autism-relevant behaviors	Three-chamber social behavior task, social olfaction, self-grooming behavior	Kelly et al., 2020
		Posterior vermis – FN – ventromedial thalamus– mPFC	Behavioral flexibility		
		DCN – intralaminar thalamic nuclei – dorsal striatum	Goal-directed behavior	T-maze test : forced alteration task (mouse)	Xiao et al., 2018
		Cerebellar vermis lobule IV/V or simplex lobule – hippocampus CA1	Cognition	Object location memory task, object recognition memory task (mouse)	Zeidler et al., 2020
		Cerebellar cortex (– thalamus, amygdala, or prelimbic cortex)	Regulation of long-term fear memory	Fear conditioning (mouse)	Han et al., 2021
		FN – parafascicular thalamus – amygdala	Anxiety	Elevated plus maze, light-dark box (mouse)	Frontera et al., 2020

*For the reference, recent studies adding new information or review articles regarding motor functions of historically studied pathways are also listed (blue). Note that this table shows functions of individual pathways demonstrated or suggested by studies, yet don’t deny possibilities of these pathways being involved also in other functions.*

## Historically Studied Efferent Pathways of the Cerebellum

**FIGURE 1:**
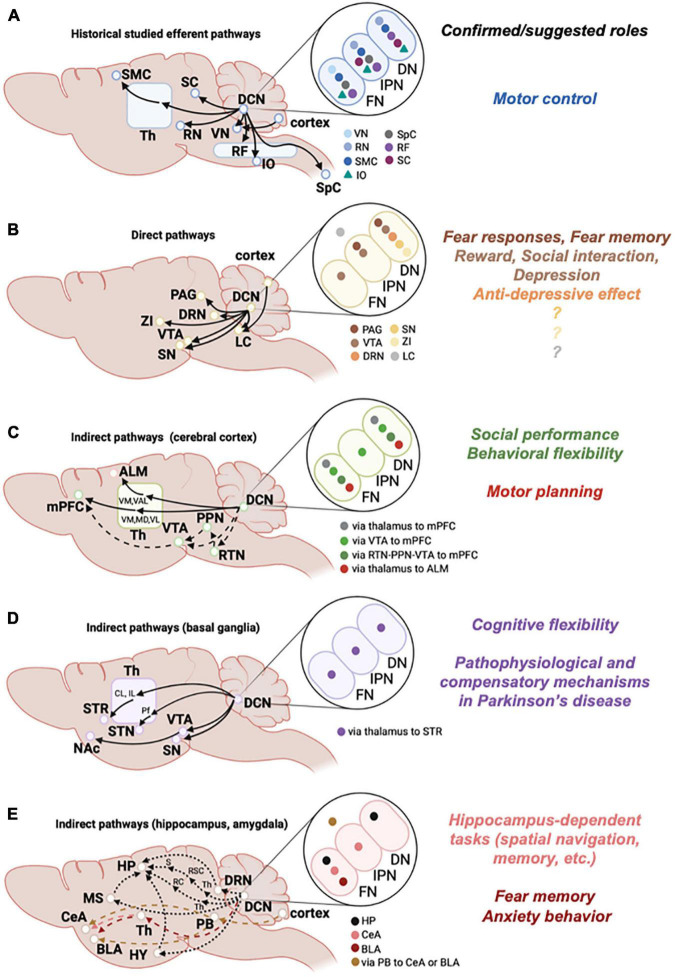
Efferent pathways from the cerebellum. Left: Schematic diagrams of historically studied efferent pathways related to motor functions **(A)**, and of direct **(B)** or indirect **(C,D)** efferent pathways that are considered to be associated with non-motor functions. Indirect pathways are further categorized according to the target brain regions, i.e., cortex **(C)**, basal ganglia **(D)**, and hippocampus or amygdala **(E)**. Solid lines are pathways that were experimentally confirmed, and dashed lines are pathways that were suggested. Brain regions written in small size of letters in E are positioned independent of the actual locations in the brain. Middle: Target brain regions that each DCN subregion connects with are shown by color-coded symbols (e.g., in A, light blue symbol is shown in the FN, but not in the IPN or DN, indicating that the vestibular nucleus (VN) receives connections from the FN, but not from the IPN or DN). Note that DCN subregions projecting to the LC **(B)** and to the amygdala via PB **(E)** are not determined, so that these symbols are shown outside of ellipses showing DCN subregions. Right: Functions of pathways that are experimentally demonstrated or suggested are summarized. Colors correspond to the pathways of target brain regions shown in the middle. Th, thalamus; RF, reticular formation; SMC, sensorimotor cortex; SpC, spinal cord; ZI, zona incerta; RTN, reticulotegmental nuclei; PPN, pedunculopontine tegmental nuclei; VM, ventromedial thalamus; MD, mediodorsal thalamus; VAL, ventral anterior lateral thalamus; VL, ventrolateral thalamus; STR, striatum; CL, centrolateral thalamus; IL, intralaminar thalamus; Pf, parafascicular thalamus; HP, hippocampus; HY, hypothalamus; MS, medial septum; RC, rhinal cortex; RSC, retrosplenial cortex; S, subiculum; PB, parabrachial nucleus; CeA, central amygdala; BLA, basolateral amygdala. See text for other abbreviations.

As is clear from cerebellar motor functions, the cerebellum has efferent pathways to regions associated with motor control (Figure 1A; Manto et al., 2012; Ruigrok and Teune, 2014). Recent studies have further advanced our understanding of the complexity or precise motor functions of these efferent pathways (Table 1), by taking advantage of historically accumulated information about these pathways and their functional relevance.

One of the long-studied cerebellar efferent pathways is the cerebellovestibular tract, an efferent pathway from the cerebellum to the vestibular nucleus in the brainstem (Walberg, 1972; Barmack, 2003; Kheradmand and Zee, 2011; Beh et al., 2017). The cerebellovestibular tract is an exceptional efferent pathway with respect to its direct projections from PCs (Sekirnjak et al., 2003), although neurons in the FN also project to the vestibular nucleus. The cerebellovestibular tract is known to be important for oculomotor control, and three cerebellar regions are known to be closely associated with oculomotor control (Kheradmand and Zee, 2011; Beh et al., 2017). One is the paraflocculus and flocculus, which control sustained pursuit of eye movements (voluntary motor activity) and vestibulo-ocular reflex (VOR) or optokinetic reflex (reflexive motor activity), respectively. The second region is the nodulus and ventral uvula (lobules IX and X of the vermis), which are responsible for low-frequency vestibular responses. The third region is the dorsal oculomotor vermis around lobules V to VII, and their target in the FN, which is involved in saccade and pursuit initiation. PCs in flocculus, paraflocculus, and lobules IX and X directly project to the vestibular nucleus, while PCs in lobules V to VII appear to have both direct and indirect projections through the FN (Kheradmand and Zee, 2011; Hashimoto et al., 2018). In addition, there is a well-known direct projection from PCs in the lateral part of the vermis to the lateral vestibular nucleus, which is known as Deiters’ nucleus, an origin of the lateral vestibulospinal tract (Voogd, 2016). Among these efferent pathways, the functions of the pathway from the flocculus to the vestibular nucleus have been frequently studied, because of its relevance to VOR, which is a reflex to stabilize gaze during head movement by moving the eyes using the vestibular system, and is recognized as an appropriate model system to analyze cerebellar motor learning (Ito, 1998). An interesting idea was proposed that VOR memory is first formed in the cerebellar cortex and then transferred to the vestibular nucleus (Shutoh et al., 2006). This idea was further supported by a recent study, which showed a temporal correlation between the transfer of intrinsic plasticity from PCs to vestibular nucleus neurons and VOR memory consolidation (Jang et al., 2020).

The red nucleus (RN) is another well-known target region of cerebellar efferent projections. The RN is located in the most rostral part of the ventral midbrain, and receives inputs from the IPN and the DN (Flumerfelt et al., 1973; Stanton, 1980; Asanuma et al., 1983; Kennedy et al., 1986). Interestingly, the RN shows a large evolutionary difference (Cacciola et al., 2019; Basile et al., 2021). The RN is divided into a caudal magnocellular part (mRN) and a rostral parvocellular part (pRN), which send projections to the spinal cord and IO, respectively (Onodera and Hicks, 2009). Whereas the RN in primitive animals is mostly composed of magnocellular-type neurons, the division of the two parts becomes clearer and the mRN tends to be smaller in primates. The mRN receives inputs from the IPN, but the pRN receives inputs from the DN (Basile et al., 2021). Consistent with the evolutionary difference, the IPN-mRN appears to be involved in motor functions. Specifically, studies have demonstrated that the pathway from the simplex lobule of the cerebellar cortex to the anterior IPN, and then to the mRN, is responsible for the delay eyeblink conditioning, which has often been used as a model system to investigate cerebellum-dependent associative motor learning (Freeman and Steinmetz, 2011). Whereas the functions of DN-pRN are not clarified, the evolutionary difference raises the possibility that the DN-pRN may be involved in higher cognitive functions.

The reticular formation includes many interconnected nuclei throughout the brainstem, and acts as a relay center for many fundamental brain functions, including somatic motor control (Mangold and Das, 2021). The DCN sends prominent projections to the reticular formation (Teune et al., 2000; Ruigrok and Teune, 2014). Three nuclei in the DCN, except for the posterior IPN, appear to contribute substantially to regulation of the pontomedullary reticular formation, which is the main source of the reticulospinal tract controlling posture and locomotion (Prentice and Drew, 2001; Stapley and Drew, 2009). In addition, a recent study demonstrated that the pathway from the cerebellar vermis to the contralateral ventral medullary reticular formation through the FN contributes to the delay eyeblink conditioning by cooperating with the canonical pathway of simplex lobule-anterior IPN-RN (Wang et al., 2020).

The thalamus is also a well-known target of DCN neurons, and mediates the communications between the cerebellum and the sensorimotor cerebral cortex to control movement (Bosch-Bouju et al., 2013; Hintzen et al., 2018). All three DCNs project to wide areas of the thalamus (Teune et al., 2000; Gornati et al., 2018; Fujita et al., 2020), although regions projected by the IPN shifted dorsolaterally and regions projected by the FN shifted ventromedially relative to the DN (Kebschull et al., 2020). In terms of cerebellar projections to the motor thalamus, such as the ventrolateral and ventromedial nuclei, which have connections with the premotor and motor cortex, motor control through the specific cerebellothalamic pathways has been continuously revealed. For example, a pathway from crus I to the ventrolateral thalamus via unidentified region of the DCN was shown to be involved in sensorimotor integration (Proville et al., 2014). A specific population of DCN neurons in the anterior IPN, which project to the caudal forelimb area of motor cortex through ventral anterior-ventral lateral thalamus, was shown to regulate the positioning and timing of forelimb movements (Low et al., 2018). Furthermore, a recent study demonstrated that the ventral anterolateral subdivision in motor thalamus was a transit point of a pathway from the IPN and the DN to the caudal forelimb area of motor cortex, and this pathway was responsible for context-dependent movement initiation (Dacre et al., 2021). The thalamic regions receiving cerebellar inputs include not only the motor thalamus, but also the intralaminar thalamus, such as the mediodorsal, parafascicular, and centrolateral nuclei (Fujita et al., 2020). Consistently, the cerebellothalamic pathways have been shown to be important also for cerebellar cognitive functions, which will be described later (see the section “Pathways From the Cerebellum to the Cerebral Cortex for Non-primary Motor Functions”).

The efferent pathway from the cerebellum to the superior colliculus (SC) has also been long known (Angaut, 1969; Batton et al., 1977; Roldán and Reinoso-Suárez, 1981; Kawamura et al., 1982). All three nuclei of the DCN appear to project to the SC in a topographically distinct manner, although the DN and IPN send stronger projections than the FN (Roldán and Reinoso-Suárez, 1981; Doykos et al., 2020). The SC is thought to be an area for sensorimotor integration to initiate motor behaviors, including eye and head movements (Ito and Feldheim, 2018), leading to the idea that the DCN-SC pathway directly controls eye and head movements, apart from the function of the cerebellovestibular tract in oculomotor control (Roldán and Reinoso-Suárez, 1981). Alternatively, considering the functions of the SC in visually guided limb movement (Courjon et al., 2004; Steinmetz et al., 2019) and the importance of the cerebellum in controlling the precision of limb positions (Becker and Person, 2019; Wagner et al., 2021), the DCN-SC pathway may provide predictive information of limb positions to successfully achieve a target, as proposed (Doykos et al., 2020). The precise motor functions of this pathway need to be determined in further studies.

In addition to the abovementioned cerebellar efferent pathways to other brain regions associated with motor control, it has been long known that the cerebellum also sends direct projections to the spinal cord [e.g. (Thomas et al., 1956; Fukushima et al., 1977; Matsushita and Hosoya, 1978; Asanuma et al., 1980; Liang et al., 2011; Wang et al., 2018)]. A study using retrograde labeling further characterized the cerebellospinal pathways, demonstrating that distinct populations of DCN neurons in the anterior IPN send ipsilateral projections to the cervical, thoracic, and lumbar cords, whereas contralateral connections from the posterior IPN and FN are limited to the cervical cord (Sathyamurthy et al., 2020). In contrast to the IPN and FN, projections from the DN to the spinal cord were not observed. The study also suggested that the direct projections from the cerebellum to the spinal cord are involved in pathway-specific, skilled motor control.

The cerebellar nucleo-olivary pathway consists of efferent projections from the DCN to the IO, and has the unique property of GABAergic projecting neurons, unlike most other efferent pathways (Bengtsson and Hesslow, 2013; Voogd et al., 2013). Interestingly, as nucleo-olivary, olivo-cortical, and corticonuclear pathways follow zonal network arrangements, a closed loop appears to be formed between the cerebellar cortex, DCN, and IO (Bengtsson and Hesslow, 2013; Chaumont et al., 2013). Thus, this nucleo-olivary pathway is thought to send feedback information to the IO, and was suggested to provide negative feedback mechanisms to block associative motor learning assessed in the eyeblink conditioning (Kim et al., 1998). A recent study demonstrated that the nucleo-olivary pathway generated negative prediction error signals that proactively trigger the extinction of associative motor memory (Kim et al., 2020). In addition, the nucleo-olivary projections have been proposed to control synchronous activity of IO neurons (Lefler et al., 2014), which are electrically coupled through gap junctions. Indeed, another recent study showed that the inhibition of nucleo-olivary projections triggered synchronous activity of IO neurons, and this was the mechanism of state changes underlying skilled movement (Wagner et al., 2021).

## New Lines of Evidence on Efferent Pathways Associated With Non-Motor Functions: Direct Pathways

Besides the abovementioned well-known efferent pathways, cerebellar efferent axons also directly project to other brain regions (Figure 1B). Although the existence of some of these pathways was identified some time ago (Teune et al., 2000), detailed investigations were not performed until recently. There are two possible reasons for this. First, the cerebellum has traditionally been thought to be important solely for motor control, yet these other brain regions receiving cerebellar projections are not primarily associated with motor functions. This may have led to the idea that, as the functional relevance of these projections is unclear, their investigations is not very important. Second, owing to the lack of appropriate techniques, it was difficult to precisely characterize or manipulate the relatively minor projecting pathways. In recent years, various lines of evidence, including accurate diagnoses in clinical studies [see, e.g. (Schmahmann and Sherman, 1998; Koziol et al., 2014; Adamaszek et al., 2017; Habas, 2021)], have suggested that the importance of the cerebellum in non-motor functions should be re-evaluated. There are now many techniques that can be used to trigger network-specific molecular expression, and cutting-edge analyses have been performed to clarify the properties of new networks and their functions. Thus, there are no reasons not to investigate these historically less well studied projecting pathways, and in fact, these direct pathways and their non-motor functions (Table 1), particularly functions associated with emotions or mental conditions, have recently been gaining attention, although they may also be involved in motor functions.

### Cerebellar Projections to the Ventral Tegmental Area

The ventral tegmental area (VTA), which is located in the midbrain, is a major sources of dopamine neurons in the brain, and is involved in a variety of brain functions (Morales and Margolis, 2017). Specifically, dopamine neurons in the VTA are best characterized as a key regulator of reward- and stress-associated behaviors (Russo and Nestler, 2013; Fox and Lobo, 2019). The VTA receives inputs from many brain regions (Zahm et al., 2011; Watabe-Uchida et al., 2012; Beier et al., 2015), and the DCN was shown to be a region projecting to the VTA (Snider et al., 1976; Parker et al., 2014; Beier et al., 2015; Carta et al., 2019). Presumably owing to the relatively minor connections, cerebellar projections to the VTA were not always detected (Teune et al., 2000). Nevertheless, sophisticated viral-genetic tracing of the input-output organization of the VTA clearly demonstrated the monosynaptic connection from the DCN to the VTA (Beier et al., 2015). Functional excitatory synaptic connections from the DCN neurons were also confirmed in not only dopamine neurons, but also GABAergic neurons in the VTA, using optogenetic stimulation of DCN neurons (Carta et al., 2019). Thus, even though anatomical connections from the DCN to the VTA appear to be minor, the VTA receives direct inputs from the DCN. It was originally suggested that projections originate from the FN and DN (Parker et al., 2014), yet a study in preprint at this moment showed using adeno-associated virus (AAV)-based circuit mapping that the DCN-VTA connections were contralateral, and originated mainly from the DN and partly from the IPN (Baek et al., 2021). The study also showed that among all the VTA regions, the dorsolateral posterior VTA was the main target of the DCN projections.

Consistent with the functional connections from the DCN to the VTA, activation of this pathway was shown to contribute to reward- and stress-associated behaviors (Figure 1B). Using optogenetic manipulation of axons of DCN neurons in the VTA, activation of this pathway was demonstrated to be rewarding and to be required for social behaviors (Carta et al., 2019). On the other hand, it was demonstrated in the abovementioned preprint study using chemogenetic manipulation of VTA-projecting DCN neurons that chronic activation of these neurons triggers depression-like behaviors (Baek et al., 2021). Although it is still unknown as to how the activation of the DCN efferent pathways projecting to the VTA can positively and negatively affect mental conditions, i.e., rewarding and depression, this may be reasonable given the heterogeneous functions of the VTA (Morales and Margolis, 2017).

Where the VTA neurons receiving inputs from the cerebellum project to is an important question. It was shown that cerebellar activation triggers dopamine release in the medial prefrontal cortex (mPFC) (Rogers et al., 2011, 2013), suggesting that the cerebellum affects emotional states via the regulation of dopamine release in the mPFC. It would be interesting to further analyze these cerebellum-VTA neuronal circuits by additional anatomical studies using transsynaptic tracing and by physiological studies measuring dopamine release and neuronal activity.

### Cerebellar Projections to the Periaqueductal Gray

The periaqueductal gray (PAG) in the midbrain takes the form of a longitudinal column (Bandler and Shipley, 1994), and receives many types of neuronal inputs (Tovote et al., 2015; George et al., 2019). The PAG is known to be associated with active and passive responses to threat (Gross and Canteras, 2012; Tovote et al., 2015; George et al., 2019), including freezing behavior, which has often been used to measure fear responses in rodent (Roelofs, 2017). Both learned and innate freezing behaviors are mediated by the activity of glutamatergic neurons in the ventrolateral PAG (vlPAG) that project to the magnocellular nucleus, and their activity is regulated by different types of inputs, such as GABAergic inputs from the amygdala and glutamatergic inputs from the prefrontal cortex (Tovote et al., 2016; Rozeske et al., 2018). The vlPAG includes not only glutamatergic neurons, but also diverse populations of neurons, including GABAergic, serotonergic, and dopaminergic neurons (Suckow et al., 2013; Taylor et al., 2019).

Anterograde tracing studies demonstrated that DCN neurons, mainly in the FN and DN, send axonal projections to the PAG (Gonzalo-Ruiz and Leichnetz, 1990; Teune et al., 2000), and direct projections from the FN to the vlPAG were further confirmed by recent studies using AAV- or retrograde tracing-based mapping (Frontera et al., 2020; Vaaga et al., 2020). DCN neurons in the FN make synapses onto diverse types of neurons in the vlPAG, because studies have demonstrated anatomical and functional synaptic connections onto glutamatergic, GABAergic, and dopaminergic neurons of the vlPAG (Frontera et al., 2020; Vaaga et al., 2020). Considering the possible involvement of the cerebellum in freezing behaviors (Supple et al., 1987, 1988; Sacchetti et al., 2002, 2004; Koutsikou et al., 2014), the identification of network connections would lead to the idea that the cerebellar regulation of vlPAG plays a role in freezing behaviors. One study supported this idea by showing that cerebellar inputs from the FN modulate dopamine interneurons in the vlPAG and in turn regulate the activity of Chx10-expressing glutamatergic neurons in the vlPAG, which reliably triggered freezing upon activation (Vaaga et al., 2020). Another study using direct chemogenetic manipulation of the FN-vlPAG pathway further demonstrated that this pathway bidirectionally regulates the strength of the fear memory formed during conditioning: increased activity weakened the memory, and decreased activity strengthened the memory (Frontera et al., 2020). As altered synaptic regulation in the cerebellum led to the enhancement of fear memory, and such cerebellum-mediated fear memory correlated with the increase in activity of the fear circuitry, in regions such as the amygdala and prefrontal cortex (Han et al., 2021), the FN-vlPAG pathway may not only regulate freezing behaviors as a fear response, but also fine tune the formation of fear memory through affecting fear circuitry (Figure 1B).

### Cerebellar Projections to the Dorsal Raphe

The dorsal raphe nucleus (DRN) is located in the midbrain and the pons, and is implicated in many functions through its projections to broad areas (Walker and Tadi, 2021). The DRN is composed of a heterogeneous population of neurons, in terms of the types of neurotransmitters, their molecular expression, and projection patterns (Huang et al., 2019; Ren et al., 2019). It is well known that the DRN is a major source of serotonin neurons, yet other neurotransmitters also play important roles in DRN functions (Liu et al., 2014; Li et al., 2016; Lin et al., 2020). One important function of the DRN is reward processing (Luo et al., 2015). Serotonergic neurons in the DRN have also been shown to have antidepressive effects (Urban et al., 2016; You et al., 2016; Nishitani et al., 2019), which are presumably linked to reward processing. The heterogeneity of the DRN also arises from the complex and diverse inputs from many brain regions, one of which is the DN (Pollak Dorocic et al., 2014; Ren et al., 2018). Although the role of the DN-DRN pathway has not been clarified, it may cooperate with the DN-VTA pathway to regulate reward- and stress-associated behaviors. Mapping studies of inputs to the DRN (Pollak Dorocic et al., 2014; Ren et al., 2018) demonstrated that the DRN receives direct inputs from the DN, but not from the FN that is innervated by PCs in the cerebellar vermis. On the other hand, electrical stimulation of the vermis of depressed animals led to their recovery from depressive symptoms and an increase in firing of DRN serotonergic neurons (Bambico et al., 2018). Considering these studies, there may be an indirect pathway from the vermis and FN to the DRN that causes antidepressive effects (Figure 1B), in addition to the direct pathway from the DN to the DRN.

### Cerebellar Projections to the Locus Coeruleus

In concurrence with dopamine and serotonin, noradrenaline is also an important neuromodulator that affects mental conditions (Ruhé et al., 2007; Nutt, 2008). The locus coeruleus (LC), which is located in the brainstem just under the cerebellum, is a major noradrenergic source in the brain. The LC-noradrenaline system is involved in a wide range of behaviors, such as arousal, attention, motivation, and stress responses (Benarroch, 2009; Eschenko et al., 2017; Poe et al., 2020; Ross and Van Bockstaele, 2020), although the dopamine release from LC neurons has also been shown to have various functions (Kempadoo et al., 2016; Takeuchi et al., 2016; Beas et al., 2018). Like other brain regions discussed in this section, the LC also receives inputs from and sends outputs to a wide variety of brain regions. Interestingly, not only DCN neurons, but also cerebellar PCs directly send axonal projections to the LC (Schwarz et al., 2015; Breton-Provencher and Sur, 2019). As the vestibular nucleus, which is another structure receiving direct inputs from PCs, is also located in the brainstem just under the cerebellum, direct PC projections might be a common feature among these brainstem regions. The LC-projecting PCs are distributed throughout the ipsilateral cerebellar vermis (Schwarz et al., 2015), and innervate both GABAergic and noradrenergic neurons in the LC (Breton-Provencher and Sur, 2019). It would be interesting to establish a technique to specifically manipulate LC-projecting PCs or LC neurons regulated by PCs, and to investigate their specific functions.

### Direct Cerebellar Projections to a Wide Variety of Other Brain Regions

In addition to the abovementioned brain regions, DCN neurons directly project to a wide variety of other brain regions, such as parafascicular thalamic nucleus, zona incerta, substantia nigra (SN), parabrachial nucleus, laterodorsal tegmental nucleus, pedunculopontine tegmental nucleus, nucleus incertus, and supramammillary region (Teune et al., 2000; Fujita et al., 2020; Kebschull et al., 2020). Although some of them will be described in the next section regarding indirect pathways, we don’t discuss many of them in this article, because their precise functions and network properties have not yet been identified. Nevertheless, we hope that these pathways will be studied in the near future. Specifically, the direct projections to the SN and the zona incerta from the DN (Teune et al., 2000; Watabe-Uchida et al., 2012; Kebschull et al., 2020) might be worth investigation, given their close association with the basal ganglia, which is another brain region responsible primarily for motor control, and is also associated with non-motor functions. As the cerebellum and the basal ganglia have indirect network connections, and their functional interactions have often been described, we will discuss them in the next section. The cerebellum is generally considered to operate prediction of motor and non-motor events (Sokolov et al., 2017; Hull, 2020) by integrating efferent copy and sensory feedback. The signals to fine-tune specific functions are likely then delivered through designated efferent projections to other brain regions responsible for the functions. Thus, projections to a broad range of brain regions may be crucial for cerebellar multifunctionality.

## New Lines of Evidence on Efferent Pathways Associated With Non-Motor Functions: Indirect Pathways

Cerebellar non-motor functions are mediated not only via the direct pathways to non-motor brain regions, but also via pathways that indirectly connect to brain regions responsible for higher cognitive functions. Similar to the abovementioned connections between the cerebellum and motor cortex, the cerebellar indirect efferent pathway for cognitive functions is expected to pass through the thalamus, considering the involvement of the thalamus in many types of cognitive functions. Although many functionally divided thalamic nuclei are thought to simply relay relevant information, lines of evidence have indicated more complex functions of the thalamus by the interplay between different thalamic nuclei and efferent pathways (Fama and Sullivan, 2015; Sherman, 2016; Halassa and Kastner, 2017; Halassa and Sherman, 2019; Wolff and Vann, 2019; Nelson, 2021). The cerebellum projects to wider areas of the thalamus than expected from cerebellar motor functions (Fujita et al., 2020; Kebschull et al., 2020), yet their functions cannot simply be described only by understanding which nuclei of the thalamus receive cerebellar efferent projections, because of the complex functions of the thalamus. It is therefore important to identify cerebellar disynaptic or polysynaptic connections to other brain regions through the thalamus and to link them with specific functions. Alternatively, it is easy to imagine that the cerebellum indirectly affects the functions of some brain regions via the abovementioned direct pathway. Indeed, several studies have demonstrated cerebellar projections to specific areas of the cerebral cortex, hippocampus, basal ganglia, and amygdala through the thalamus or VTA, and have identified their non-primary motor functions, as described in this section.

### Pathways From the Cerebellum to the Cerebral Cortex for Non-primary Motor Functions

The mPFC is implicated in various cognitive functions, such as emotional control, motivation, fear extinction, sociability, decision making, and long-term and short-term memory (Euston et al., 2012; Grossmann, 2013; Peters et al., 2013; Domenech and Koechlin, 2015; Giustino and Maren, 2015; Xu et al., 2019). Given such a wide range of cognitive functions, an idea was proposed that the general role of the mPFC is to resolve conflicting responses (Euston et al., 2012; Peters et al., 2013), presumably by taking into account positive and negative information carried by broad types of inputs, and providing contextually appropriate neuronal signals. The interaction between the cerebellum and the mPFC has been reported in several studies, including studies recording synchronized oscillations in rodents and functional connectomics analyses in humans or primates (Ramnani, 2006; Kalmbach et al., 2009; Chen et al., 2016; Stoodley et al., 2017; McAfee et al., 2019), the latter of which suggested connections in the direction from the cerebellum to the mPFC. Experiments in rodents showed that electrical stimulation of the FN evoked local-field potentials in the mPFC (Watson et al., 2014), implying that cerebellar outputs functionally connect to the mPFC. Anatomical connections from the DN to the mPFC through the thalamus were also demonstrated in primates using retrograde transneuronal transport of herpes simplex virus type 1 (Middleton and Strick, 2001). Based on these studies, it has become clear that cerebellar neurons indirectly project to the mPFC through the thalamus, in addition to oppositely directed connections from the mPFC to the cerebellum through the pontine nucleus (Kalmbach et al., 2009). A recent study demonstrated functions of the cerebellar-mPFC pathway on behaviors associated with autism spectrum disorders (ASDs) (Kelly et al., 2020). Specifically, two cerebellar efferent pathways to the mPFC were found to be responsible for the various phenotypes observed in cerebellar dysfunction-dependent ASDs: a pathway from crus I in the cerebellar cortex to the DN, and another pathway from the cerebellar posterior vermis to the FN, converging on the ventromedial thalamus projecting to the mPFC, which were involved in the regulation of social performance and behavioral flexibility, respectively.

The cerebellum-dependent regulation of mPFC may be in part through dopamine release, because the electrical stimulation of lobule IV/V PCs and the DN were shown to evoke dopamine release in the mPFC (Mittleman et al., 2008). Two possibilities were discussed in this previous paper regarding pathways from the DCN that regulate dopamine release in the mPFC. The first is the pathway from the DN or IPN to the VTA through the reticulotegmental nuclei in the pons, and to the pedunculopontine tegmental nuclei. The second is the pathway from the DCN to the mediodorsal and ventrolateral thalamic nuclei, via which glutamate neurons control dopamine release by regulating presynaptic terminals in the mPFC. As DCN neurons directly project to the VTA (Snider et al., 1976; Parker et al., 2014; Beier et al., 2015; Carta et al., 2019; Baek et al., 2021), this direct projection may in turn regulate dopamine release in the mPFC. All pathways shown or suggested to date may mediate cerebellar-mPFC circuits (Figure 1C), and regulation through these various pathways appears to be reasonable, considering the functions of the mPFC, which require broad types of inputs.

The motor cortex can be divided into the primary and premotor areas. The primary motor cortex, which is a well-known region that receives connections from the cerebellum through the thalamus, is thought to have a predominant role in motor execution. The anterolateral motor cortex (ALM) in mice is thought to be equivalent to the premotor cortex in primates, and to be involved in motor planning by presenting preparatory activity that is crucial for subsequent proper movements (Guo et al., 2014; Li et al., 2015). In addition to the primary motor cortex, functions of cerebellar projections to the ALM have recently been identified, in an effort to understand cerebellar cognitive functions. Studies have shown that optogenetic excitation of the DCN alters preparatory activity in the ALM, indicating the importance of cerebellar-ALM pathways in motor planning (Gao et al., 2018; Chabrol et al., 2019). However, two studies showed different pathways working in motor planning, either from the FN or from the DN to the ALM (Figure 1C). Axons of DCN neurons from the FN and the DN were mainly present in the ventral medial nucleus and the ventral anterior-lateral nucleus of the thalamus, respectively. Both nuclei overlapped with thalamic regions that projected to the ALM, although overlapping regions of axons from the FN were much wider than those of axons from the DN (Gao et al., 2018). As suggested in a previous study (Chabrol et al., 2019), further analysis is required to solve questions as to whether these efferent projections work separately on motor planning in different behavioral paradigms, or actually cooperate with each other.

The connections between the cerebellum and the cerebral cortex are often bidirectional, involving the cortico-cerebellar pathway through the pontine nucleus and the cerebello-cortical pathway through the thalamus (Kelly and Strick, 2003; Krienen and Buckner, 2009; Buckner et al., 2011; Proville et al., 2014; Léna and Popa, 2016; Palesi et al., 2017; Brissenden et al., 2018; Gao et al., 2018). Intriguingly, not only bidirectional connections, but also closed-loop circuits are formed, which are composed of networks of the cerebellar regions projecting to the cerebral cortical regions that then project back to the same cerebellar regions (Proville et al., 2014; Gao et al., 2018). These closed-loop circuits may be effective for the precise adjustment or amplification of neuronal signals, and indeed, the sensorimotor cortico-cerebellar loop and the premotor cortico-cerebellar loop were shown to be important for fine movement control and for persistent preparatory activity, respectively (Proville et al., 2014; Gao et al., 2018).

### Pathways From the Cerebellum to the Basal Ganglia

The basal ganglia are a group of subcortical nuclei, which generally include the striatum consisting of the caudate and putamen, the internal (GPi) and external (GPe) segments of globus pallidus, the substantia nigra pars reticulata (SNr) and pars compacta (SNc), and the subthalamic nucleus (STN) (Lanciego et al., 2012; Purves et al., 2018). The basal ganglia can be broadly categorized into three nuclei, namely, the input nucleus of the striatum, the intrinsic nuclei of the GPe, SNc, and STN, and the output nuclei of the GPi and SNr. As with the cerebellum, the basal ganglia have also been implicated in motor control. Although both the cerebellum and basal ganglia have connections with the motor cortex, they are traditionally thought to have distinct roles in movement, with the cerebellum involved in real-time fine tuning of movement, and the basal ganglia involved in the production of action command (Doya, 2000), by projecting to different thalamic nuclei. This view has gradually been revised, mainly from three aspects. First, outputs from the cerebellum and the basal ganglia are not completely segregated, but interact with each other in the thalamus (Bosch-Bouju et al., 2013; Hintzen et al., 2018). Second, roles of both the cerebellum and the basal ganglia are not limited to motor control, but extend to cognitive functions (Doya, 2000; Middleton and Strick, 2000; Bostan and Strick, 2018; Pierce and Péron, 2020). Third, whereas interactions between the cerebellum and the basal ganglia are generally considered to be through the cerebral cortex, their reciprocal connections that do not include the cerebral cortex have also been identified (Bostan and Strick, 2018). In particular, there may be several pathways in the direction from the cerebellum to the basal ganglia (Figure 1D, next paragraph).

The disynaptic anatomical connections from the cerebellum to the basal ganglia were first demonstrated in rodents by observation of the overlap and synaptic contacts in the central lateral nucleus of the thalamus, between anterogradely labeled DN neuron axons and retrogradely labeled neurons projecting to the striatum (Ichinohe et al., 2000). Similar connections were then confirmed in primates by the transneuronal transport of rabies virus (Hoshi et al., 2005). Considering the time required for transneuronal transport, cerebellar outputs, partially from the IPN and FN, but mostly from the DN, disynaptically project to the putamen in the striatum presumably through the thalamus, including the central lateral nucleus. The connections from the DN to the striatum through the intralaminar thalamic nuclei, including the central lateral nucleus, were also functionally confirmed in rodents (Chen et al., 2014). In this previous study, a short latency of activity modulation was detected in the striatum upon the electrical or optogenetic stimulation of the DN, and the modulation was no longer detected when the intralaminar thalamus was inhibited. Importantly, the cerebello-striatal network through the thalamus was shown to be involved in the cognitive flexibility observed in mice performing a striatum-dependent reward-driven task (Xiao et al., 2018).

In addition to the connections from the DCN to the striatum through the intralaminar thalamus, other pathways are also likely to contribute to the functional interaction between the cerebellum and the basal ganglia. A possibility was recently proposed that the DCN makes disynaptic connections with the STN through the parafascicular nucleus of the thalamus (Watson et al., 2021). Because the nucleus accumbens (NAc) has sometimes been considered as a part of the basal ganglia based on its projections to the GPe and SN (Salgado and Kaplitt, 2015), one of the cerebellar-basal ganglia pathways may be from the DCN to the NAc through the VTA, of which projection to the NAc has been well characterized (Russo and Nestler, 2013). Although its functions are not yet determined, the direct connections from the DCN to the SN (Teune et al., 2000; Watabe-Uchida et al., 2012) may be involved in the cerebellar regulation of the basal ganglia. Considering that the SN can be categorized as an output nucleus of the basal ganglia, and that, as described here, there are several possible pathways from the cerebellum to the basal ganglia, the cerebellum may regulate the basal ganglia in a variety of ways (Figure 1D). It has been suggested that the cerebellum is involved in both pathophysiological and compensatory mechanisms of Parkinson’s disease (Martinu and Monchi, 2013), and such bidirectional involvement might be reasonable, considering that there are many pathways, through which the cerebellum regulates the basal ganglia.

### Pathways From the Cerebellum to the Hippocampus

The hippocampus is one of the most studied regions of the brain, and is well known to be crucial for learning, memory, and spatial navigation (Eichenbaum, 2004; Bird and Burgess, 2008). Even though connections from the cerebellum to the hippocampus were suggested a long time ago by electrophysiological recordings from the hippocampus upon cerebellar stimulation, and by observation of degenerating axons in the hippocampus after damage to the FN (Heath and Harper, 1974; Snider and Maiti, 1976; Heath et al., 1978; Newman and Reza, 1979), anatomical connections have only recently been reported. Studies using viral vector-based circuit tracing showed disynaptic and trisynaptic connections from the cerebellum to the hippocampus (Bohne et al., 2019; Watson et al., 2019), and specifically, tracing using a retrograde transneuronal property of rabies virus suggested several pathways (Figure 1E). Although the precise pathways between the DCN and the dentate gyrus in the hippocampus still need to be identified, vermal lobule VI and crus I of the cerebellar cortex appear to be regions that affect the hippocampus through the FN and the DN, respectively (Watson et al., 2019). Functional implications of cerebellar-hippocampal circuit connections have also been analyzed recently. Alterations of cerebellar activity were detected during hippocampus-dependent tasks (Iglói et al., 2015; Yu and Krook-Magnuson, 2015; Babayan et al., 2017). Cerebellar-specific gene manipulation and optogenetic excitation affected hippocampus-dependent behaviors (Rochefort et al., 2013; Lefort et al., 2019; Zeidler et al., 2020). It would be interesting to identify the precise cerebellar-hippocampal pathway, as this would enable us to specifically manipulate the pathway and subsequently identify its specific functions.

### Pathways From the Cerebellum to the Amygdala

Amygdala is well known to play a role in the expression of fear and in the processing of fear-associated signals (Ehrlich et al., 2009; Ressler, 2010). As described in the section on direct efferent pathways, accumulating lines of evidence indicate the involvement of the cerebellum in the presentation of fear responses and the formation of fear memory (Sacchetti et al., 2002, 2004; Moreno-Rius, 2018; Ernst et al., 2019; Vaaga et al., 2020; Han et al., 2021). Consistent with the functional relevance, connections between the cerebellum and the amygdala were suggested in functional magnetic resonance imaging studies in human subjects (Dean et al., 2014; Nicholson et al., 2015; Leutgeb et al., 2016). Connections from the cerebellum to the amygdala were also demonstrated in rodents by functional analyses. Activation of the FN or anterior IPN resulted in the inhibition of central amygdala activity (Magal and Mintz, 2014), and the cerebellum-mediated enhancement of fear memory was shown to correlate with the activity increase in the amygdala (Han et al., 2021). Anatomical pathways were then suggested, in which the cerebellar FN could project to the basolateral amygdala through the parafascicular thalamus (Figure 1E; Frontera et al., 2020). Alternatively, because the parabrachial nucleus receives projections directly from PCs and indirectly through the FN (Hashimoto et al., 2018), and projects to the amygdala (Saper and Loewy, 1980; Jhamandas et al., 1996; Cai et al., 2018), the efferent pathways to the amygdala might be mediated via the parabrachial nucleus. Considering the potential of motor training as a therapy for abnormal fear responses (Faria et al., 2018; Tanner et al., 2018), the cerebellar-amygdala pathways may be worthy of further investigation.

## Techniques to Unravel the Complexity of Cerebellar Efferent Pathways

In general, anatomical tracing techniques using specific labeling lead to a better understanding of neuronal networks, and such techniques, which are readily available, have been continuously developed. Techniques used for the investigation of cerebellar efferent pathways have also been changing gradually with time (Table 2). In addition to anatomical tracing, physiological analyses have been used to detect functional connections from the cerebellum to other brain regions (Table 2). Furthermore, advanced techniques have been beneficial to test functions of specific efferent pathways on a behavioral level. In this section, we summarize the techniques that have been used to understand cerebellar efferent pathways. Details of the individual pathways, including currently controversial or inconsistent results regarding specific pathways, have been described above, and example pathways that have been studied utilizing these techniques are also listed in Table 2.

**TABLE 2:** A summary of techniques that have been used for studies of cerebellar efferent pathways.

	Techniques		Methods	Examples		
				Species/transgenic mouse	Pathways	References
Anatomical tracing	Fink-Heimer method		Observation of degenerated axons after lesion of the DCN or the cerebellum	Rabbit Cat	DN – reticular formation Cerebellum – VTA, SN	Tang et al., 1987 Snider et al., 1976
	Autoradiography		Tracing using radiolabeled amino acid	Rat	DCN – thalamus	Angaut et al., 1985b
	HRP Neurohistochemical technique		Tracing using HRP and following staining	Cat Cat	DCN – SC DCN – SC	Kawamura et al., 1982 Roldán and Reinoso-Suárez, 1981
	Anterograde or retrograde tracer conjugated with dyes		Fluorogold Biotinylated dextran amine Cholera toxin subunit B, Retrobeads	Mouse Rat Mouse Mouse Mouse	FN – reticular formation DCN – other brain regions Collateral of DCN neurons – cerebellar cortex FN – vlPAG FN – vlPAG	Lu et al., 2013 Teune et al., 2000 Houck and Person, 2015 Frontera et al., 2020 Vaaga et al., 2020
	Viral vector-based labeling	Anterograde tracing	AAV AAV with cell-type specific expression system	Mouse Mouse (Vglut2-Cre) Mouse (GAD-Cre, GlyT2-Cre) Mouse (GAD2-Cre) Mouse (Sox14-Cre) Mouse (PV-Cre)	FN – vlPAG FN – vlPAG DCN – cerebellar cortex DCN – IO DCN – IO DCN – gigantocellular reticular nucleus	Vaaga et al., 2020 Frontera et al., 2020 Ankri et al., 2015 Lefler et al., 2014 Prekop et al., 2018 Zhou M. et al., 2020
		Retrograde tracing	rAAV2-retro, CAV2	Mouse Mouse Mouse Mouse	Cerebellum – parabrachial nucleus DCN – spinal cord DCN – vlPAG DCN – VTA	Hashimoto et al., 2018 Sathyamurthy et al., 2020 Frontera et al., 2020 Baek et al., 2021
		Disynaptic labeling	Glycoprotein-deleted pseudotyped rabies AAV expressing WGA-Cre AAV serotype1	Mouse Mouse (Vglut2-Cre, GAD2-Cre) Mouse (tdTomato^+/+^) Mouse Mouse (Ai14)	Cerebellar cortex – DCN – thalamus –striatum IPN, DN – SC DCN – thalamus – hippocampus DCN – thalamus –striatum DCN – thalamus – mPFC	Xiao et al., 2018 Doykos et al., 2020 Bohne et al., 2019 Xiao et al., 2018 Kelly et al., 2020
		Polysynaptic labeling	Rabies virus Herpes simplex virus strain (H129)	Mouse Mouse	Cerebellum – several regions – hippocampus Cerebellum – several regions – cortex	Watson et al., 2019 Pisano et al., 2021
		TRIO, cTRIO		Mouse (DAT-Cre, GAD2-Cre) Mouse (Dbh-Cre) Mouse (Sert-Cre)	DCN – VTA Cerebellum – LC DCN – DRN	Beier et al., 2015 Schwarz et al., 2015 Ren et al., 2018
	Comprehensive circuit analysis with gene expression profiling		Characterization of DCN neurons by projection mapping and single cell gene expression analysis (qPCR, RNA sequencing)	Mouse Mouse, Chicken, Human donors	FN output circuits DCN (mainly DN) output circuits	Fujita et al., 2020 Kebschull et al., 2020
Functional connections	Optogenetic manipulation and activity recording		Applying photoactivation (e.g., ChR2) or photoinhibition (e.g., ArchT, HR) to the cerebellum, while recording activity in target brain regions by silicon probes (SP), microelectrode (ME), electroencephalogram (EEG), calcium imaging using GCaMP (Ca), or immunohistochemistry (IH)	Mouse (PCP2-Cre, Ai32) Mouse (PV-Cre, Ai32, Ai39) Mouse (PCP2-ChR2-YFP) Mouse Mouse (PCP2-Cre, Ai32) Mouse (PCP2-Cre, Ai32) Mouse Mouse Mouse (PCP2-Cre)	Cerebellum – ALM (SP) Cerebellum –hippocampus (EEG) Cerebellum – thalamus -motor cortex (ME) Cerebellum – VTA (ME) Cerebellum – ALM (SP) Cerebellum –hippocampus (ME and Ca) Cerebellum – motor cortex (SP) Cerebellum – vlPAG (ME) Cerebellum – several regions – cortex (IH)	Gao et al., 2018 Krook-Magnuson et al., 2014 Proville et al., 2014 Carta et al., 2019 Chabrol et al., 2019 Zeidler et al., 2020 Dacre et al., 2021 Frontera et al., 2020 Pisano et al., 2021
	Electrical manipulation and activity recording		Bipolar electrode stimulation onto the cerebellum, while recording activity by microelectrode in target regions	Rat	Cerebellum – DRN Cerebellum – mPFC	Bambico et al., 2018
	Chemogenetic manipulation and activity measurement		Chemogenetic drug administration in mice having cell-type specific expression of chemogenetic molecules, and microelectrode recording (ME) or immunohistochemistry (IH) in target regions	Mouse (PCP2-Cre) Mouse (Vglut2-Cre)	Cerebellum – mPFC (ME) Cerebellum – striatum (IH)	Kelly et al., 2020 Xiao et al., 2018
	Ex vivo electro-physiological recording		Whole cell patch clamp recording in slices of target brain regions with optogenetic stimulation on DCN neuron axons	Mouse Mouse Mouse Mouse Mouse	Cerebellum – VTA Cerebellum – VTA Cerebellum – thalamus Cerebellum – vlPAG DCN – parafascicular thalamic nucleus	Carta et al., 2019 Baek et al., 2021 Gornati et al., 2018 Vaaga et al., 2020 Xiao et al., 2018
Behavioral functions of specific efferent pathways	Optogenetic manipulation of DCN neuron axons in target brain regions		Expression of optogenetic molecules in DCN neurons by injecting AAV, and light application onto target brain regions during behavioral tests	Mouse Mouse Mouse	Cerebellum – VTA Cerebellum – IO Cerebellum – thalamus -motor cortex	Carta et al., 2019 Kim et al., 2020 Dacre et al., 2021
	Chemogenetic or optogenetic manipulation through molecular expression in specific efferent pathways		Expression of chemogenetic or optogenetic molecules in DCN neurons specifically projecting to target brain regions by utilizing rAAV2-retro or CAV2 expressing Cre, and their manipulation during behavioral tests	Mouse Mouse Mouse Mouse	Cerebellum – spinal cord Cerebellum – vlPAG Cerebellum – VTA Cerebellum – IO	Sathyamurthy et al., 2020 Frontera et al., 2020 Baek et al., 2021 Wagner et al., 2021
	Chemogenetic or optogenetic manipulation of disynaptic cerebellar efferent pathways		Expression of chemogenetic or optogenetic molecules in thalamic neurons receiving inputs from the cerebellum, and manipulation of thalamic axon terminals by light or drug application in target brain regions during behavioral tests	Mouse Mouse	Cerebellum – thalamus – striatum Cerebellum – thalamus – mPFC	Xiao et al., 2018 Kelly et al., 2020

*This table also includes examples of efferent pathways that were demonstrated by studies using the techniques. Note that types of transgenic mice are shown in this table, in case if the specific techniques were used in combination with the transgenic mice. Vglut, vesicular glutamate transporter; GAD, glutamic acid decarboxylase; GlyT, glycine transporter; Sox, Sex-determining region Y-related high-mobility-group box; PV, parvalbumin; DAT, dopamine transpoter; Dbh, dopamine b-hydroxylase; Sert, serotonin transporter; PCP2, Purkinje cell protein 2. See text for other abbreviations.*

### Techniques Used in Early Studies on Cerebellar Efferent Pathways

In early studies, cerebellar efferent projections were tested using the Fink-Heimer method (Fink and Heimer, 1967) in animals with lesions in the DCN (Snider et al., 1976; Faull and Carman, 1978; Tang et al., 1987), in which degenerated nerve terminals of DCN neurons could be observed in target regions. Autoradiography was also used after DCN injection with radiolabeled amino acids that can be taken up by neurons (Angaut et al., 1985a,b). Although there may have been potential concerns about the observation being under unphysiological or toxic conditions, these studies suggested the importance of performing further research regarding cerebellar network connections. Horseradish peroxidase (HRP) was used as a less toxic retrograde tracer (Roldán and Reinoso-Suárez, 1981; Kawamura et al., 1982), although the efficiency of neuronal uptake was relatively low (Saleeba et al., 2019). In recent years, anterograde and retrograde tracers have been developed, improved, and frequently used owing to their convenience: tracers without serious safety concerns are commercially available and have sufficiently strong signals. The injection of anterograde tracers into the DCN increased our understanding of the overall projection patterns from the DCN (Teune et al., 2000). On the other hand, the injection of retrograde tracers into a target region resulted in the labeling of a specific group of DCN neurons (Lu et al., 2013; Houck and Person, 2015; Frontera et al., 2020; Vaaga et al., 2020). Even though tracers are convenient tools, we also need to understand their drawbacks (Saleeba et al., 2019). One point to consider is that the specificity of labeling basically relies on their localized injection.

### Anatomical Mapping Analyses Using Viral Vector-Based Labeling

Viral vector-based labeling has become a basic method for studies on the anatomical properties of neuronal circuits (Sarno and Robison, 2018; Haggerty et al., 2020; Liu et al., 2020; Xu et al., 2020). Unlike chemical tracers, viral vectors generally enable cell type- or network-specific labeling, by combining multiple viral injections or the use of transgenic mice with the expression of Cre recombinase (Cre). Given the heterogeneous properties of DCN neurons, investigations using specific labeling provide an accurate understanding of cerebellar efferent networks. Indeed, network property analyses in a cell type-specific manner were performed by injecting AAV with a Cre-dependent cassette into the DCN of cell type-specific Cre transgenic mice (Lefler et al., 2014; Ankri et al., 2015; Prekop et al., 2018; Zhou M. et al., 2020). Some studies also used viral vectors demonstrating retrograde transport, such as AAVs engineered to have efficient retrograde access [rAAV2-retro, (Tervo et al., 2016)] or a canine adenoviral vector [CAV2 (Del Rio et al., 2019)], to label projection-specific DCN neurons (Hashimoto et al., 2018; Frontera et al., 2020; Sathyamurthy et al., 2020; Baek et al., 2021). To observe disynaptic connections from the DCN, anterograde transsynaptic tracing was also performed (Xiao et al., 2018; Bohne et al., 2019; Kelly et al., 2020) by injecting recombinase-expressing AAV serotype 1 with transsynaptic properties (Zingg et al., 2017) or AAV expressing Cre fused with wheat germ agglutinin (WGA) (Gradinaru et al., 2010) into the DCN (Xiao et al., 2018; Bohne et al., 2019; Kelly et al., 2020). In these experiments, Cre- or flippase-dependent molecular expression was usually triggered by AAV injection into the intermediate region or by usage of reporter mice. Moreover, polysynaptic connections from the cerebellum were analyzed using the retrograde and anterograde transneuronal viral tracers, rabies virus (RV) and herpes simplex virus strain H129, respectively, which enabled dissection of the complex connections from the cerebellum to the hippocampus or the neocortex (Watson et al., 2019; Pisano et al., 2021).

Advanced anatomical analyses have the potential to increase our understanding of cerebellar efferent neuronal circuits. Systematic analyses of single-cell gene expression and anatomical projection mapping have characterized heterogeneous DCN neurons (Fujita et al., 2020; Kebschull et al., 2020). The results of these studies indicate that DCN neurons are subdivided into finer groups than expected, and individual groups at least in part have their own functions through their projections. These results share common traits with the traditional idea that the three nuclei of the DCN have different functions, yet greatly advanced our understanding of the cerebellar efferent pathways from the DCN. A tracing technique using a combination of a viral-genetic tool, named TRIO, was developed to analyze input and output organizations, and this sophisticated analysis confirmed previously unappreciated cerebellar projections into the neuromodulatory system, and further demonstrated the region of projection through the disynaptic pathways (Beier et al., 2015; Schwarz et al., 2015; Ren et al., 2018).

### Analyses to Detect Functional Connections From the Cerebellum to Other Brain Regions

In addition to anatomical investigations, neuronal network connections between the cerebellum and other brain regions have been functionally tested by several techniques, including viral vector-based expression of optogenetic molecules. The most direct measurement method of functional connections is electrophysiological recording from target regions during electrical, optogenetic, or chemogenetic manipulation of cerebellar activity (Krook-Magnuson et al., 2014; Proville et al., 2014; Bambico et al., 2018; Gao et al., 2018; Carta et al., 2019; Chabrol et al., 2019; Frontera et al., 2020; Kelly et al., 2020; Zeidler et al., 2020; Dacre et al., 2021). The immunohistochemical analysis of activity-dependent molecules in target regions can be also used to detect activity changes in these regions after cerebellar manipulation (Xiao et al., 2018; Zeidler et al., 2020; Pisano et al., 2021). Alterations of molecular expression patterns specifically in the cerebellum may disrupt the functional integrity of the cerebellum, and in turn result in activity changes in other brain regions (Kelly et al., 2020; Rudolph et al., 2020; Han et al., 2021). Activity changes in these studies denote that the cerebellum has functional effects on the recorded brain regions through direct or indirect network connections. The direct functional connections have been verified by recording synaptic transmission from the neurons in target brain regions *ex vivo*, upon the photostimulation of channelrhodopsin-expressing DCN neuronal terminals (Gornati et al., 2018; Xiao et al., 2018; Carta et al., 2019; Vaaga et al., 2020; Baek et al., 2021).

### Methods Used to Identify Functions of Specific Cerebellar Efferent Pathways on a Behavioral Level

Optogenetic and chemogenetic tools are beneficial to understand the roles of specific neuronal circuits in many types of brain functions and dysfunctions [e.g. (Muir et al., 2019; Biselli et al., 2021)]. Studies on the functions of cerebellar efferent pathways on a behavioral level have also benefited from these tools. In particular, optogenetic molecules were expressed in DCN neurons by the injection of AAV into the DCN, and photostimulation was applied to the target brain regions, which enabled the manipulation of specific cerebellar efferent pathways during behavioral analyses, and thus leading to an understanding of their functions (Carta et al., 2019; Kim et al., 2020; Dacre et al., 2021). The manipulation of specific cerebellar efferent pathways can also be achieved by specific molecular expression using a combination of viral vector injections. In these experiments, the abovementioned rAAV-retro or CAV2 expressing Cre was injected into target brain regions, and AAVs triggering Cre-dependent expression of chemogenetic or optogenetic molecules were injected into the DCN, which resulted in the specific expression of molecules in the DCN neurons projecting to the target brain regions. The manipulation of these neurons during behavioral tests by chemogenetic drug administration or photostimulation of the DCN clarified the functions of these efferent pathways (Frontera et al., 2020; Sathyamurthy et al., 2020; Baek et al., 2021; Wagner et al., 2021). Furthermore, disynaptically connected cerebellar efferent pathways were also investigated, as follows: AAV serotype 1 expressing Cre, or AAV expressing WGA-Cre was injected into the DCN, and AAV triggering the Cre-dependent expression of chemogenetic or optogenetic molecules was injected into the intermediate regions, which resulted in the expression of these molecules specifically in neurons of the intermediate regions receiving inputs from the DCN. The functions of disynaptic efferent pathways were then tested by specifically applying a chemogenetic drug or by photostimulation to the target brain regions during the behavioral analyses (Xiao et al., 2018; Kelly et al., 2020).

## Conclusion and Perspectives

In any field of neuroscience, technical innovations by the development of cutting-edge tools, equipment, or techniques are often crucial not only for a better understanding or new findings of a particular issue, but also for opening new directions in the field or reevaluating underappreciated areas. Although the cerebellum is a brain region with a long history of study, research on neuronal networks emerging from the cerebellum have benefited from these technical innovations. Indeed, several efferent pathways covered in this review article were already proposed decades ago, but have recently been clarified or revised, and have been found to be involved in particular functions, by taking advantage of these new techniques. Owing to these clarification and findings, the cerebellum is now considered to have multiple brain functions through projections to many other brain regions. To facilitate further the studies on cerebellar multifunctionality and a wide variety of efferent pathways, we would like to propose four research directions as a next step.

(1) Further clarification of efferent pathways: As described above (see the section “Direct Cerebellar Projections to a Wide Variety of Other Brain Regions”), in addition to the target brain regions discussed in this article, DCN neurons directly project to many other brain regions, yet the precise connective pathways have not been identified. Pathways of indirect projections from the cerebellum to the mPFC, hippocampus, and amygdala have not been completely clarified, and there may be other crucial regions that are polysynaptically affected by these cerebellar projections. It may be possible to clarify these pathways by tracing analyses using the appropriate labeling techniques, such as transsynaptic labeling or a combination of retrograde and anterograde labeling.
(2) Understanding of the collateral projections of DCN neurons: Individual DCN neurons are known to project to different brain regions through collateral axons (Kebschull et al., 2020), and their collateralizing properties may be characterized by whole brain mapping of collateral axons after target region-specific labeling of DCN neurons, as was previously done for DCN neurons projecting to the zona incerta and reticular nucleus (Kebschull et al., 2020).
(3) Information integration through the cerebellum and efferent pathways: Even though there is the idea that different domains within the cerebellum are responsible for different functions, considering the distribution of receptive fields that is cerebellar regions responding to sensory stimulation of different body parts (Manni and Petrosini, 2004; Apps and Hawkes, 2009), functional domains appear to be intermingled and DCN neurons may in turn handle the integrated information. Indeed, a recent study demonstrated that a small population of neurons in the anterior IPN is responsible for motor coordination of multiple body parts (Heiney et al., 2021). A possibility is that the integrated information is distributed to several different regions through the collateral axons of DCN neurons, and thus it will be interesting to test the function of collateral axons by specifically manipulating some of them, in addition to clarifying the properties of the collateral axons, as described in (2).
(4) Motor and non-motor functions of cerebellar efferent pathways: The information integration described in (3) may also be applicable to the information associated with motor and non-motor functions. This suggests that the recently demonstrated efferent pathways, which we discussed in this article from the aspect of non-primary motor functions (Table 1), may also be associated with motor functions, and historically studied pathways may inversely be associated with non-motor functions. Alternatively, the activity of cerebellar efferent pathways may be affected by a wider range of input signals than expected. The functions of individual efferent pathways will need to be systematically clarified by a variety of behavioral analyses, with the specific manipulation of these pathways.

In summary, we comprehensively summarized cerebellar efferent pathways and their functions in this article, mainly from the aspect of non-motor cerebellar functions. The highly heterogeneous properties of efferent pathways in the cerebellum appear to be reasonable, considering their multiple functions. Thus, toward understanding cerebellar multifunctionality, it is important to further investigate the properties and functions of individual efferent pathways, and to comprehensively interpret various lines of evidence. For such investigation, further technological innovations and the establishment of creative experimental or analytic strategies are thought to be necessary.

## Author Contributions

SK and SJ wrote the manuscript draft and made the figures. YY and KT-Y conceptualized, wrote the manuscript draft, and corrected the manuscript. SB and HP edited the manuscript and figures. All authors contributed to the article and approved the submitted version.

## Conflict of Interest

The authors declare that the research was conducted in the absence of any commercial or financial relationships that could be construed as a potential conflict of interest.

## Publisher’s Note

All claims expressed in this article are solely those of the authors and do not necessarily represent those of their affiliated organizations, or those of the publisher, the editors and the reviewers. Any product that may be evaluated in this article, or claim that may be made by its manufacturer, is not guaranteed or endorsed by the publisher.

## References

[B1] Adamaszek M. D’Agata F. Ferrucci R. Habas C. Keulen S. Kirkby K. C. (2017). Consensus paper: cerebellum and emotion. *Cerebellum* 16 552–576. 10.1007/s12311-016-0815-8 27485952

[B2] Aizenman C. D. Huang E. J. Linden D. J. (2003). Morphological correlates of intrinsic electrical excitability in neurons of the deep cerebellar nuclei. *J. Neurophysiol.* 89 1738–1747. 10.1152/jn.01043.2002 12686564

[B3] Angaut P. (1969). The fastigio-tectal projections. An anatomical experimental study. *Brain Res.* 13 186–189. 10.1016/0006-8993(69)90155-3 5806138

[B4] Angaut P. Cicirata F. Pantò M. R. (1985a). An autoradiographic study of the cerebellopontine projections from the interposed and lateral cerebellar nuclei in the rat. *J. Hirnforsch.* 26 463–470. 4067284

[B5] Angaut P. Cicirata F. Serapide F. (1985b). Topographic organization of the cerebellothalamic projections in the rat. An autoradiographic study. *Neuroscience* 15 389–401. 10.1016/0306-4522(85)90221-0 4022330

[B6] Ankri L. Husson Z. Pietrajtis K. Proville R. Léna C. Yarom Y. (2015). A novel inhibitory nucleo-cortical circuit controls cerebellar Golgi cell activity. *Elife* 4:e06262. 10.7554/eLife.06262.015 PMC4461794 25965178

[B7] Apps R. Hawkes R. (2009). Cerebellar cortical organization: a one-map hypothesis. *Nat. Rev. Neurosci.* 10 670–681. 10.1038/nrn2698 19693030

[B8] Asanuma C. Thach W. R. Jones E. G. (1983). Anatomical evidence for segregated focal groupings of efferent cells and their terminal ramifications in the cerebellothalamic pathway of the monkey. *Brain Res.* 286 267–297. 10.1016/0165-0173(83)90016-4 6189562

[B9] Asanuma H. Larsen K. Yumiya H. (1980). Peripheral input pathways to the monkey motor cortex. *Exp. Brain Res.* 38 349–355. 10.1007/BF00236655 6245903

[B10] Babayan B. M. Watilliaux A. Viejo G. Paradis A. L. Girard B. Rondi-Reig L. (2017). A hippocampo-cerebellar centred network for the learning and execution of sequence-based navigation. *Sci. Rep.* 7:17812. 10.1038/s41598-017-18004-7 29259243 PMC5736633

[B11] Baek S. J. Park J. Kim J. Yamamoto Y. Tanaka-Yamamoto K. (2021). VTA-projecting cerebellar neurons mediate stress-dependent depression-like behavior. *bioRxiv* [Preprint] 10.1101/2021.08.25.457606 PMC8843095 35156922

[B12] Bagnall M. W. Zingg B. Sakatos A. Moghadam S. H. Zeilhofer H. U. du Lac S. (2009). Glycinergic projection neurons of the cerebellum. *J. Neurosci.* 29 10104–10110. 10.1523/JNEUROSCI.2087-09.2009 19675244 PMC3196611

[B13] Bambico F. R. Comai S. Diwan M. Hasan S. M. N. Conway J. D. Darvish-Ghane S. (2018). High frequency stimulation of the anterior vermis modulates behavioural response to chronic stress: involvement of the prefrontal cortex and dorsal raphe? *Neurobiol. Dis.* 116 166–178. 10.1016/j.nbd.2018.03.011 29727711

[B14] Bandler R. Shipley M. T. (1994). Columnar organization in the midbrain periaqueductal gray: modules for emotional expression? *Trends Neurosci.* 17 379–389. 10.1016/0166-2236(94)90047-7 7817403

[B15] Barmack N. H. (2003). Central vestibular system: vestibular nuclei and posterior cerebellum. *Brain Res. Bull.* 60 511–541. 10.1016/S0361-9230(03)00055-8 12787870

[B16] Basile G. A. Quartu M. Bertino S. Serra M. P. Boi M. Bramanti A. (2021). Red nucleus structure and function: from anatomy to clinical neurosciences. *Brain Struct. Funct.* 226 69–91. 10.1007/s00429-020-02171-x 33180142 PMC7817566

[B17] Batton R. R. III Jayaraman A. Ruggiero D. Carpenter M. B. (1977). Fastigial efferent projections in the monkey: an autoradiographic study. *J. Comp. Neurol.* 174 281–305. 10.1002/cne.901740206 68041

[B18] Baumel Y. Jacobson G. A. Cohen D. (2009). Implications of functional anatomy on information processing in the deep cerebellar nuclei. *Front. Cell Neurosci.* 3:14. 10.3389/neuro.03.014.2009 19949453 PMC2783015

[B19] Beas B. S. Wright B. J. Skirzewski M. Leng Y. Hyun J. H. Koita O. (2018). The locus coeruleus drives disinhibition in the midline thalamus via a dopaminergic mechanism. *Nat. Neurosci.* 21 963–973. 10.1038/s41593-018-0167-4 29915192 PMC6035776

[B20] Becker M. I. Person A. L. (2019). Cerebellar control of reach kinematics for endpoint precision. *Neuron* 103 335–348.e335. 10.1016/j.neuron.2019.05.007 31174960 PMC6790131

[B21] Beckinghausen J. Sillitoe R. V. (2019). Insights into cerebellar development and connectivity. *Neurosci. Lett.* 688 2–13. 10.1016/j.neulet.2018.05.013 29746896 PMC6222004

[B22] Beekhof G. C. Osório C. White J. J. van Zoomeren S. van der Stok H. Xiong B. (2021). Differential spatiotemporal development of Purkinje cell populations and cerebellum-dependent sensorimotor behaviors. *Elife* 10:e63668. 10.7554/eLife.63668.sa2 PMC8195607 33973524

[B23] Beh S. C. Frohman T. C. Frohman E. M. (2017). Cerebellar control of eye movements. *J. Neuroophthalmol.* 37 87–98. 10.1097/WNO.0000000000000456 27643747

[B24] Beier K. T. Steinberg E. E. DeLoach K. E. Xie S. Miyamichi K. Schwarz L. (2015). Circuit architecture of VTA dopamine neurons revealed by systematic input-output mapping. *Cell* 162 622–634. 10.1016/j.cell.2015.07.015 26232228 PMC4522312

[B25] Benarroch E. E. (2009). The locus ceruleus norepinephrine system: functional organization and potential clinical significance. *Neurology* 73 1699–1704. 10.1212/WNL.0b013e3181c2937c 19917994

[B26] Bengtsson F. Hesslow G. (2013). “Feedback control in the olivo-cerebellar loop,” in *Handbook Of The Cerebellum And Cerebellar Disorders*, eds Manto M. Schmahmann J. D. Rossi F. Gruol D. L. Koibuchi N. (Dordrecht: Springer), 1079–1099. 10.1007/978-94-007-1333-8_45

[B27] Bird C. M. Burgess N. (2008). The hippocampus and memory: insights from spatial processing. *Nat. Rev. Neurosci.* 9 182–194. 10.1038/nrn2335 18270514

[B28] Biselli T. Lange S. S. Sablottny L. Steffen J. Walther A. (2021). Optogenetic and chemogenetic insights into the neurocircuitry of depression-like behaviour: a systematic review. *Eur. J. Neurosci.* 53 9–38. 10.1111/ejn.14603 31633833

[B29] Bohne P. Schwarz M. K. Herlitze S. Mark M. D. (2019). A new projection from the deep cerebellar nuclei to the hippocampus via the ventrolateral and laterodorsal thalamus in mice. *Front. Neural Circuits* 13:51. 10.3389/fncir.2019.00051 31447652 PMC6695568

[B30] Bosch-Bouju C. Hyland B. I. Parr-Brownlie L. C. (2013). Motor thalamus integration of cortical, cerebellar and basal ganglia information: implications for normal and parkinsonian conditions. *Front. Comput. Neurosci.* 7:163. 10.3389/fncom.2013.00163 24273509 PMC3822295

[B31] Bostan A. C. Strick P. L. (2018). The basal ganglia and the cerebellum: nodes in an integrated network. *Nat. Rev. Neurosci.* 19 338–350. 10.1038/s41583-018-0002-7 29643480 PMC6503669

[B32] Breton-Provencher V. Sur M. (2019). Active control of arousal by a locus coeruleus GABAergic circuit. *Nat. Neurosci.* 22 218–228. 10.1038/s41593-018-0305-z 30643295 PMC6385895

[B33] Brissenden J. A. Tobyne S. M. Osher D. E. Levin E. J. Halko M. A. Somers D. C. (2018). Topographic cortico-cerebellar networks revealed by visual attention and working memory. *Curr. Biol.* 28 3364–3372. 10.1016/j.cub.2018.08.059 30344119 PMC6257946

[B34] Buckner R. L. Krienen F. M. Castellanos A. Diaz J. C. Yeo B. T. (2011). The organization of the human cerebellum estimated by intrinsic functional connectivity. *J. Neurophysiol.* 106 2322–2345. 10.1152/jn.00339.2011 21795627 PMC3214121

[B35] Cacciola A. Milardi D. Basile G. A. Bertino S. Calamuneri A. Chillemi G. (2019). The cortico-rubral and cerebello-rubral pathways are topographically organized within the human red nucleus. *Sci. Rep.* 9:12117. 10.1038/s41598-019-48164-7 31431648 PMC6702172

[B36] Cai Y. Q. Wang W. Paulucci-Holthauzen A. Pan Z. Z. (2018). Brain circuits mediating opposing effects on emotion and pain. *J. Neurosci.* 38 6340–6349. 10.1523/JNEUROSCI.2780-17.2018 29941444 PMC6041794

[B37] Canto C. B. Witter L. De Zeeuw C. I. (2016). Whole-cell properties of cerebellar nuclei neurons *in vivo*. *PLoS One* 11:e0165887. 10.1371/journal.pone.0165887 27851801 PMC5112928

[B38] Carta I. Chen C. H. Schott A. L. Dorizan S. Khodakhah K. (2019). Cerebellar modulation of the reward circuitry and social behavior. *Science* 363:eaav0581. 10.1126/science.aav0581 30655412 PMC6711161

[B39] Cerminara N. L. Lang E. J. Sillitoe R. V. Apps R. (2015). Redefining the cerebellar cortex as an assembly of non-uniform Purkinje cell microcircuits. *Nat. Rev. Neurosci.* 16 79–93. 10.1038/nrn3886 25601779 PMC4476393

[B40] Chabrol F. P. Blot A. Mrsic-Flogel T. D. (2019). Cerebellar contribution to preparatory activity in motor neocortex. *Neuron* 103 506–519. 10.1016/j.neuron.2019.05.022 31201123 PMC6693889

[B41] Chaumont J. Guyon N. Valera A. M. Dugué G. P. Popa D. Marcaggi P. (2013). Clusters of cerebellar Purkinje cells control their afferent climbing fiber discharge. *Proc. Natl. Acad. Sci. U.S.A.* 110 16223–16228. 10.1073/pnas.1302310110 24046366 PMC3791757

[B42] Chen C. H. Fremont R. Arteaga-Bracho E. E. Khodakhah K. (2014). Short latency cerebellar modulation of the basal ganglia. *Nat. Neurosci.* 17 1767–1775. 10.1038/nn.3868 25402853 PMC4241171

[B43] Chen H. Wang Y. J. Yang L. Sui J. F. Hu Z. A. Hu B. (2016). Theta synchronization between medial prefrontal cortex and cerebellum is associated with adaptive performance of associative learning behavior. *Sci. Rep.* 6:20960. 10.1038/srep20960 26879632 PMC4754690

[B44] Courjon J. H. Olivier E. Pélisson D. (2004). Direct evidence for the contribution of the superior colliculus in the control of visually guided reaching movements in the cat. *J. Physiol.* 556 675–681. 10.1113/jphysiol.2004.061713 15020693 PMC1665002

[B45] Czubayko U. Sultan F. Thier P. Schwarz C. (2001). Two types of neurons in the rat cerebellar nuclei as distinguished by membrane potentials and intracellular fillings. *J. Neurophysiol.* 85 2017–2029. 10.1152/jn.2001.85.5.2017 11353018

[B46] Dacre J. Colligan M. Clarke T. Ammer J. J. Schiemann J. Chamosa-Pino V. (2021). A cerebellar-thalamocortical pathway drives behavioral context-dependent movement initiation. *Neuron* 109 2326–2338.e2328. 10.1016/j.neuron.2021.05.016 34146469 PMC8315304

[B47] De Zeeuw C. I. Ten Brinke M. M. (2015). Motor Learning and the Cerebellum. *Cold Spring Harb. Perspect. Biol.* 7:a021683. 10.1101/cshperspect.a021683 26330521 PMC4563713

[B48] De Zeeuw C. I. Simpson J. I. Hoogenraad C. C. Galjart N. Koekkoek S. K. Ruigrok T. J. (1998). Microcircuitry and function of the inferior olive. *Trends Neurosci.* 21 391–400. 10.1016/S0166-2236(98)01310-1 9735947

[B49] Dean A. C. Kohno M. Hellemann G. London E. D. (2014). Childhood maltreatment and amygdala connectivity in methamphetamine dependence: a pilot study. *Brain Behav.* 4 867–876. 10.1002/brb3.289 25365801 PMC4178299

[B50] Del Rio D. Beucher B. Lavigne M. Wehbi A. Gonzalez Dopeso-Reyes I. Saggio I. (2019). CAV-2 vector development and gene transfer in the central and peripheral nervous systems. *Front. Mol. Neurosci.* 12:71. 10.3389/fnmol.2019.00071 30983967 PMC6449469

[B51] Domenech P. Koechlin E. (2015). Executive control and decision-making in the prefrontal cortex. *Curr. Opin. Behav. Sci.* 1 101–106. 10.1016/j.cobeha.2014.10.007

[B52] Doya K. (2000). Complementary roles of basal ganglia and cerebellum in learning and motor control. *Curr. Opin. Neurobiol.* 10 732–739. 10.1016/S0959-4388(00)00153-7 11240282

[B53] Doykos T. K. Gilmer J. I. Person A. L. Felsen G. (2020). Monosynaptic inputs to specific cell types of the intermediate and deep layers of the superior colliculus. *J. Comp. Neurol.* 528 2254–2268. 10.1002/cne.24888 32080842 PMC8032550

[B54] Eccles J. C. Ito M. Szentágothai J. (1967). *The Cerebellum as a Neuronal Machine.* Berlin: Springer.

[B55] Ehrlich I. Humeau Y. Grenier F. Ciocchi S. Herry C. Lüthi A. (2009). Amygdala inhibitory circuits and the control of fear memory. *Neuron* 62 757–771. 10.1016/j.neuron.2009.05.026 19555645

[B56] Eichenbaum H. (2004). Hippocampus: cognitive processes and neural representations that underlie declarative memory. *Neuron* 44 109–120. 10.1016/j.neuron.2004.08.028 15450164

[B57] Ernst T. M. Brol A. E. Gratz M. Ritter C. Bingel U. Schlamann M. (2019). The cerebellum is involved in processing of predictions and prediction errors in a fear conditioning paradigm. *Elife* 8:e46831. 10.7554/eLife.46831.024 PMC6715348 31464686

[B58] Eschenko O. Mello-Carpes P. B. Hansen N. (2017). New insights into the role of the locus coeruleus-noradrenergic system in memory and perception dysfunction. *Neural Plast.* 2017:4624171. 10.1155/2017/4624171 29270321 PMC5706083

[B59] Euston D. R. Gruber A. J. McNaughton B. L. (2012). The role of medial prefrontal cortex in memory and decision making. *Neuron* 76 1057–1070. 10.1016/j.neuron.2012.12.002 23259943 PMC3562704

[B60] Fama R. Sullivan E. V. (2015). Thalamic structures and associated cognitive functions: relations with age and aging. *Neurosci. Biobehav. Rev.* 54 29–37. 10.1016/j.neubiorev.2015.03.008 25862940 PMC4457546

[B61] Faria R. S. Bereta A. L. B. Reis G. H. T. Santos L. B. B. Pereira M. S. G. Cortez P. J. O. (2018). Effects of swimming exercise on the extinction of fear memory in rats. *J. Neurophysiol.* 120 2649–2653. 10.1152/jn.00586.2018 30230992

[B62] Faull R. L. Carman J. B. (1978). The cerebellofugal projections in the brachium conjunctivum of the rat I. The contralateral ascending pathway. *J. Comp. Neurol.* 178 495–517. 10.1002/cne.901780307 19626723

[B63] Fink R. P. Heimer L. (1967). Two methods for selective silver impregnation of degenerating axons and their synaptic endings in the central nervous system. *Brain Res.* 4 369–374. 10.1016/0006-8993(67)90166-7 4166480

[B64] Flumerfelt B. A. Otabe S. Courville J. (1973). Distinct projections to the red nucleus from the dentate and interposed nuclei in the monkey. *Brain Res.* 50 408–414. 10.1016/0006-8993(73)90742-7 4196194

[B65] Fox M. E. Lobo M. K. (2019). The molecular and cellular mechanisms of depression: a focus on reward circuitry. *Mol. Psychiatry* 24 1798–1815. 10.1038/s41380-019-0415-3 30967681 PMC6785351

[B66] Freeman J. H. Steinmetz A. B. (2011). Neural circuitry and plasticity mechanisms underlying delay eyeblink conditioning. *Learn. Mem.* 18 666–677. 10.1101/lm.2023011 21969489 PMC3861981

[B67] Frontera J. L. Baba Aissa H. Sala R. W. Mailhes-Hamon C. Georgescu I. A. Léna C. (2020). Bidirectional control of fear memories by cerebellar neurons projecting to the ventrolateral periaqueductal grey. *Nat. Commun.* 11:5207. 10.1038/s41467-020-18953-0 33060630 PMC7566591

[B68] Fujita H. Kodama T. du Lac S. (2020). Modular output circuits of the fastigial nucleus for diverse motor and nonmotor functions of the cerebellar vermis. *Elife* 9:e58613. 10.7554/eLife.58613.sa2 PMC7438114 32639229

[B69] Fukushima K. Peterson B. W. Uchino Y. Coulter J. D. Wilson V. J. (1977). Direct fastigiospinal fibers in the cat. *Brain Res.* 126 538–542. 10.1016/0006-8993(77)90604-7 861735

[B70] Gao Z. Davis C. Thomas A. M. Economo M. N. Abrego A. M. Svoboda K. (2018). A cortico-cerebellar loop for motor planning. *Nature* 563 113–116. 10.1038/s41586-018-0633-x 30333626 PMC6212318

[B71] Gao Z. Proietti-Onori M. Lin Z. Ten Brinke M. M. Boele H. J. Potters J. W. (2016). Excitatory cerebellar nucleocortical circuit provides internal amplification during associative conditioning. *Neuron* 89 645–657. 10.1016/j.neuron.2016.01.008 26844836 PMC4742536

[B72] George D. T. Ameli R. Koob G. F. (2019). Periaqueductal gray sheds light on dark areas of psychopathology. *Trends Neurosci.* 42 349–360. 10.1016/j.tins.2019.03.004 30955857

[B73] Giustino T. F. Maren S. (2015). The role of the medial prefrontal cortex in the conditioning and extinction of fear. *Front. Behav. Neurosci.* 9:298. 10.3389/fnbeh.2015.00298 26617500 PMC4637424

[B74] Gonzalo-Ruiz A. Leichnetz G. R. (1990). Connections of the caudal cerebellar interpositus complex in a new world monkey (*Cebus apella*). *Brain Res. Bull.* 25 919–927. 10.1016/0361-9230(90)90189-7 2289174

[B75] Gornati S. V. Schäfer C. B. Eelkman Rooda O. H. J. Nigg A. L. De Zeeuw C. I. Hoebeek F. E. (2018). Differentiating cerebellar impact on thalamic nuclei. *Cell Rep.* 23 2690–2704. 10.1016/j.celrep.2018.04.098 29847799 PMC5990493

[B76] Gradinaru V. Zhang F. Ramakrishnan C. Mattis J. Prakash R. Diester I. (2010). Molecular and cellular approaches for diversifying and extending optogenetics. *Cell* 141 154–165. 10.1016/j.cell.2010.02.037 20303157 PMC4160532

[B77] Gross C. T. Canteras N. S. (2012). The many paths to fear. *Nat. Rev. Neurosci.* 13 651–658. 10.1038/nrn3301 22850830

[B78] Grossmann T. (2013). The role of medial prefrontal cortex in early social cognition. *Front. Hum. Neurosci.* 7:340. 10.3389/fnhum.2013.00340 23847509 PMC3701864

[B79] Guo Z. V. Li N. Huber D. Ophir E. Gutnisky D. Ting J. T. (2014). Flow of cortical activity underlying a tactile decision in mice. *Neuron* 81 179–194. 10.1016/j.neuron.2013.10.020 24361077 PMC3984938

[B80] Habas C. (2021). Functional connectivity of the cognitive cerebellum. *Front. Syst. Neurosci.* 15:642225. 10.3389/fnsys.2021.642225 33897382 PMC8060696

[B81] Haggerty D. L. Grecco G. G. Reeves K. C. Atwood B. (2020). Adeno-associated viral vectors in neuroscience research. *Mol. Ther. Methods Clin. Dev.* 17 69–82. 10.1016/j.omtm.2019.11.012 31890742 PMC6931098

[B82] Halassa M. M. Kastner S. (2017). Thalamic functions in distributed cognitive control. *Nat. Neurosci.* 20 1669–1679. 10.1038/s41593-017-0020-1 29184210

[B83] Halassa M. M. Sherman S. M. (2019). Thalamocortical circuit motifs: a general framework. *Neuron* 103 762–770. 10.1016/j.neuron.2019.06.005 31487527 PMC6886702

[B84] Han J. K. Kwon S. H. Kim Y. G. Choi J. Kim J. I. Lee Y. S. (2021). Ablation of STAT3 in Purkinje cells reorganizes cerebellar synaptic plasticity in long-term fear memory network. *Elife* 10:e63291. 10.7554/eLife.63291 33459594 PMC7813544

[B85] Hashimoto M. Yamanaka A. Kato S. Tanifuji M. Kobayashi K. Yaginuma H. (2018). Anatomical evidence for a direct projection from purkinje cells in the mouse cerebellar vermis to medial parabrachial nucleus. *Front. Neural Circuits* 12:6. 10.3389/fncir.2018.00006 29467628 PMC5808303

[B86] Heath R. G. Harper J. W. (1974). Ascending projections of the cerebellar fastigial nucleus to the hippocampus, amygdala, and other temporal lobe sites: evoked potential and histological studies in monkeys and cats. *Exp. Neurol.* 45 268–287. 10.1016/0014-4886(74)90118-6 4422320

[B87] Heath R. G. Dempesy C. W. Fontana C. J. Myers W. A. (1978). Cerebellar stimulation: effects on septal region, hippocampus, and amygdala of cats and rats. *Biol. Psychiatry* 13 501–529. 728506

[B88] Heiney S. A. Wojaczynski G. J. Medina J. F. (2021). Action-based organization of a cerebellar module specialized for predictive control of multiple body parts. *Neuron* 109 2981–2994.e2985. 10.1016/j.neuron.2021.08.017 34534455 PMC8513160

[B89] Hintzen A. Pelzer E. A. Tittgemeyer M. (2018). Thalamic interactions of cerebellum and basal ganglia. *Brain Struct. Funct.* 223 569–587. 10.1007/s00429-017-1584-y 29224175

[B90] Hoshi E. Tremblay L. Féger J. Carras P. L. Strick P. L. (2005). The cerebellum communicates with the basal ganglia. *Nat. Neurosci.* 8 1491–1493. 10.1038/nn1544 16205719

[B91] Houck B. D. Person A. L. (2015). Cerebellar premotor output neurons collateralize to innervate the cerebellar cortex. *J. Comp. Neurol.* 523 2254–2271. 10.1002/cne.23787 25869188 PMC4537674

[B92] Huang K. W. Ochandarena N. E. Philson A. C. Hyun M. Birnbaum J. E. Cicconet M. (2019). Molecular and anatomical organization of the dorsal raphe nucleus. *Elife* 8:e46464. 10.7554/eLife.46464.032 PMC6726424 31411560

[B93] Hull C. (2020). Prediction signals in the cerebellum: beyond supervised motor learning. *Elife* 9:e54073. 10.7554/eLife.54073 32223891 PMC7105376

[B94] Ichinohe N. Mori F. Shoumura K. (2000). A di-synaptic projection from the lateral cerebellar nucleus to the laterodorsal part of the striatum via the central lateral nucleus of the thalamus in the rat. *Brain Res.* 880 191–197. 10.1016/S0006-8993(00)02744-X 11033006

[B95] Iglói K. Doeller C. F. Paradis A. L. Benchenane K. Berthoz A. Burgess N. (2015). Interaction between hippocampus and cerebellum crus I in sequence-based but not place-based navigation. *Cereb. Cortex* 25 4146–4154. 10.1093/cercor/bhu132 24947462 PMC4886832

[B96] Ito M. (1998). Cerebellar learning in the vestibulo–ocular reflex. *Trends Cogn. Sci.* 2 313–321. 10.1016/S1364-6613(98)01222-4 21227227

[B97] Ito S. Feldheim D. A. (2018). The mouse superior colliculus: an emerging model for studying circuit formation and function. *Front. Neural Circuits* 12:10. 10.3389/fncir.2018.00010 29487505 PMC5816945

[B98] Jang D. C. Shim H. G. Kim S. J. (2020). Intrinsic plasticity of cerebellar purkinje cells contributes to motor memory consolidation. *J. Neurosci.* 40 4145–4157. 10.1523/JNEUROSCI.1651-19.2020 32295816 PMC7244189

[B99] Jhamandas J. H. Petrov T. Harris K. H. Vu T. Krukoff T. L. (1996). Parabrachial nucleus projection to the amygdala in the rat: electrophysiological and anatomical observations. *Brain Res. Bull.* 39 115–126. 10.1016/0361-9230(95)02084-5 8846113

[B100] Judd E. N. Lewis S. M. Person A. L. (2021). Diverse inhibitory projections from the cerebellar interposed nucleus. *Elife* 10:e66231. 10.7554/eLife.66231.sa2 PMC8483738 34542410

[B101] Kalmbach B. E. Ohyama T. Kreider J. C. Riusech F. Mauk M. D. (2009). Interactions between prefrontal cortex and cerebellum revealed by trace eyelid conditioning. *Learn. Mem.* 16 86–95. 10.1101/lm.1178309 19144967 PMC2632850

[B102] Kawamura S. Hattori S. Higo S. Matsuyama T. (1982). The cerebellar projections to the superior colliculus and pretectum in the cat: an autoradiographic and horseradish peroxidase study. *Neuroscience* 7 1673–1689. 10.1016/0306-4522(82)90026-4 7121831

[B103] Kebschull J. M. Richman E. B. Ringach N. Friedmann D. Albarran E. Kolluru S. S. (2020). Cerebellar nuclei evolved by repeatedly duplicating a conserved cell-type set. *Science* 370:eabd5059. 10.1126/science.abd5059 33335034 PMC8510508

[B104] Kelly E. Meng F. Fujita H. Morgado F. Kazemi Y. Rice L. C. (2020). Regulation of autism-relevant behaviors by cerebellar-prefrontal cortical circuits. *Nat. Neurosci.* 23 1102–1110. 10.1038/s41593-020-0665-z 32661395 PMC7483861

[B105] Kelly R. M. Strick P. L. (2003). Cerebellar loops with motor cortex and prefrontal cortex of a nonhuman primate. *J. Neurosci.* 23 8432–8444. 10.1523/JNEUROSCI.23-23-08432.2003 12968006 PMC6740694

[B106] Kempadoo K. A. Mosharov E. V. Choi S. J. Sulzer D. Kandel E. R. (2016). Dopamine release from the locus coeruleus to the dorsal hippocampus promotes spatial learning and memory. *Proc. Natl. Acad. Sci. U.S.A.* 113 14835–14840. 10.1073/pnas.1616515114 27930324 PMC5187750

[B107] Kennedy P. R. Gibson A. R. Houk J. C. (1986). Functional and anatomic differentiation between parvicellular and magnocellular regions of red nucleus in the monkey. *Brain Res.* 364 124–136. 10.1016/0006-8993(86)90993-5 3947959

[B108] Kheradmand A. Zee D. S. (2011). Cerebellum and ocular motor control. *Front. Neurol.* 2:53. 10.3389/fneur.2011.00053 21909334 PMC3164106

[B109] Kim J. J. Krupa D. J. Thompson R. F. (1998). Inhibitory cerebello-olivary projections and blocking effect in classical conditioning. *Science* 279 570–573. 10.1126/science.279.5350.570 9438852

[B110] Kim O. A. Ohmae S. Medina J. F. (2020). A cerebello-olivary signal for negative prediction error is sufficient to cause extinction of associative motor learning. *Nat. Neurosci.* 23 1550–1554. 10.1038/s41593-020-00732-1 33169031 PMC7686232

[B111] Koutsikou S. Crook J. J. Earl E. V. Leith J. L. Watson T. C. Lumb B. M. (2014). Neural substrates underlying fear-evoked freezing: the periaqueductal grey-cerebellar link. *J. Physiol.* 592 2197–2213. 10.1113/jphysiol.2013.268714 24639484 PMC4027863

[B112] Koziol L. F. Budding D. Andreasen N. D’Arrigo S. Bulgheroni S. Imamizu H. (2014). Consensus paper: the cerebellum’s role in movement and cognition. *Cerebellum* 13 151–177. 10.1007/s12311-013-0511-x 23996631 PMC4089997

[B113] Krienen F. M. Buckner R. L. (2009). Segregated fronto-cerebellar circuits revealed by intrinsic functional connectivity. *Cereb. Cortex* 19 2485–2497. 10.1093/cercor/bhp135 19592571 PMC2742600

[B114] Krook-Magnuson E. Szabo G. G. Armstrong C. Oijala M. Soltesz I. (2014). Cerebellar directed optogenetic intervention inhibits spontaneous hippocampal seizures in a mouse model of temporal lobe epilepsy. *eNeuro* 1 10.1523/ENEURO.0005-14.2014 [Epub ahead of print]. 25599088 PMC4293636

[B115] Lanciego J. L. Luquin N. Obeso J. A. (2012). Functional neuroanatomy of the basal ganglia. *Cold Spring Harb. Perspect. Med.* 2:a009621. 10.1101/cshperspect.a009621 23071379 PMC3543080

[B116] Lefler Y. Yarom Y. Uusisaari M. Y. (2014). Cerebellar inhibitory input to the inferior olive decreases electrical coupling and blocks subthreshold oscillations. *Neuron* 81 1389–1400. 10.1016/j.neuron.2014.02.032 24656256

[B117] Lefort J. M. Vincent J. Tallot L. Jarlier F. De Zeeuw C. I. Rondi-Reig L. (2019). Impaired cerebellar Purkinje cell potentiation generates unstable spatial map orientation and inaccurate navigation. *Nat. Commun.* 10:2251. 10.1038/s41467-019-09958-5 31113954 PMC6529420

[B118] Léna C. Popa D. (2016). “Cerebrocerebellar loops in the rodent brain,” in *The Neuronal Codes Of The Cerebellum*, ed. Heck D. H. (New York, NY: Elsevier), 135–153. 10.1016/B978-0-12-801386-1.00006-X

[B119] Leutgeb V. Wabnegger A. Leitner M. Zussner T. Scharmüller W. Klug D. (2016). Altered cerebellar-amygdala connectivity in violent offenders: a resting-state fMRI study. *Neurosci. Lett.* 610 160–164. 10.1016/j.neulet.2015.10.063 26523791

[B120] Li N. Chen T. W. Guo Z. V. Gerfen C. R. Svoboda K. (2015). A motor cortex circuit for motor planning and movement. *Nature* 519 51–56. 10.1038/nature14178 25731172

[B121] Li Y. Zhong W. Wang D. Feng Q. Liu Z. Zhou J. (2016). Serotonin neurons in the dorsal raphe nucleus encode reward signals. *Nat. Commun.* 7:10503. 10.1038/ncomms10503 26818705 PMC4738365

[B122] Liang H. Paxinos G. Watson C. (2011). Projections from the brain to the spinal cord in the mouse. *Brain Struct. Funct.* 215 159–186. 10.1007/s00429-010-0281-x 20936329

[B123] Lin R. Liang J. Wang R. Yan T. Zhou Y. Liu Y. (2020). The raphe dopamine system controls the expression of incentive memory. *Neuron* 106 498–514. 10.1016/j.neuron.2020.02.009 32145184

[B124] Liu Y. Hegarty S. Winter C. Wang F. He Z. (2020). Viral vectors for neuronal cell type-specific visualization and manipulations. *Curr. Opin. Neurobiol.* 63 67–76. 10.1016/j.conb.2020.03.011 32344323 PMC7484153

[B125] Liu Z. Zhou J. Li Y. Hu F. Lu Y. Ma M. (2014). Dorsal raphe neurons signal reward through 5-HT and glutamate. *Neuron* 81 1360–1374. 10.1016/j.neuron.2014.02.010 24656254 PMC4411946

[B126] Low A. Y. T. Thanawalla A. R. Yip A. K. K. Kim J. Wong K. L. L. Tantra M. (2018). Precision of discrete and rhythmic forelimb movements requires a distinct neuronal subpopulation in the interposed anterior nucleus. *Cell Rep.* 22 2322–2333. 10.1016/j.celrep.2018.02.017 29490269

[B127] Lu H. Zou Q. Gu H. Raichle M. E. Stein E. A. Yang Y. (2012). Rat brains also have a default mode network. *Proc. Natl. Acad. Sci. U.S.A.* 109 3979–3984. 10.1073/pnas.1200506109 22355129 PMC3309754

[B128] Lu L. Cao Y. Tokita K. Heck D. H. Boughter J. D. Jr. (2013). Medial cerebellar nuclear projections and activity patterns link cerebellar output to orofacial and respiratory behavior. *Front. Neural Circuits* 7:56. 10.3389/fncir.2013.00056 23565078 PMC3613706

[B129] Luo M. Zhou J. Liu Z. (2015). Reward processing by the dorsal raphe nucleus: 5-HT and beyond. *Learn. Mem.* 22 452–460. 10.1101/lm.037317.114 26286655 PMC4561406

[B130] Magal A. Mintz M. (2014). Inhibition of the amygdala central nucleus by stimulation of cerebellar output in rats: a putative mechanism for extinction of the conditioned fear response. *Eur. J. Neurosci.* 40 3548–3555. 10.1111/ejn.12714 25185877

[B131] Mangold S. A. Das J. M. (2021). *Neuroanatomy, Reticular Formatio.* Treasure Island, FL: StatPearls Publishing LLC. 32310562

[B132] Manni E. Petrosini L. (2004). A century of cerebellar somatotopy- a debated representation. *Nat. Rev. Neurosci.* 5 241–249. 10.1038/nrn1347 14976523

[B133] Manto M. Bower J. M. Conforto A. B. Delgado-García J. M. da Guarda S. N. Gerwig M. (2012). Consensus paper: roles of the cerebellum in motor control–the diversity of ideas on cerebellar involvement in movement. *Cerebellum* 11 457–487. 10.1007/s12311-011-0331-9 22161499 PMC4347949

[B134] Martinu K. Monchi O. (2013). Cortico-basal ganglia and cortico-cerebellar circuits in Parkinson’s disease: pathophysiology or compensation? *Behav. Neurosci.* 127 222–236. 10.1037/a0031226 23244290

[B135] Matsushita M. Hosoya Y. (1978). The location of spinal projection neurons in the cerebellar nuclei (cerebellospinal tract neurons) of the cat. A study with the horseradish peroxidase technique. *Brain Res.* 142 237–248. 10.1016/0006-8993(78)90633-9 630384

[B136] McAfee S. S. Liu Y. Sillitoe R. V. Heck D. H. (2019). Cerebellar lobulus simplex and crus I differentially represent phase and phase difference of prefrontal cortical and hippocampal oscillations. *Cell Rep.* 27 2328–2334. 10.1016/j.celrep.2019.04.085 31116979 PMC6538275

[B137] Middleton F. A. Strick P. L. (2000). Basal ganglia and cerebellar loops: motor and cognitive circuits. *Brain Res. Brain Res. Rev.* 31 236–250. 10.1016/S0165-0173(99)00040-5 10719151

[B138] Middleton F. A. Strick P. L. (2001). Cerebellar projections to the prefrontal cortex of the primate. *J. Neurosci.* 21 700–712. 10.1523/JNEUROSCI.21-02-00700.2001 11160449 PMC6763818

[B139] Mittleman G. Goldowitz D. Heck D. H. Blaha C. D. (2008). Cerebellar modulation of frontal cortex dopamine efflux in mice: relevance to autism and schizophrenia. *Synapse* 62 544–550. 10.1002/syn.20525 18435424 PMC3854870

[B140] Morales M. Margolis E. B. (2017). Ventral tegmental area: cellular heterogeneity, connectivity and behaviour. *Nat. Rev. Neurosci.* 18 73–85. 10.1038/nrn.2016.165 28053327

[B141] Moreno-Rius J. (2018). The cerebellum in fear and anxiety-related disorders. *Prog. Neuropsychopharmacol. Biol. Psychiatry* 85 23–32. 10.1016/j.pnpbp.2018.04.002 29627508

[B142] Moreno-Rius J. (2019). The cerebellum under stress. *Front. Neuroendocrinol.* 54:100774. 10.1016/j.yfrne.2019.100774 31348932

[B143] Muir J. Lopez J. Bagot R. C. (2019). Wiring the depressed brain: optogenetic and chemogenetic circuit interrogation in animal models of depression. *Neuropsychopharmacology* 44 1013–1026. 10.1038/s41386-018-0291-6 30555161 PMC6461994

[B144] Nelson A. J. D. (2021). The anterior thalamic nuclei and cognition: a role beyond space? *Neurosci. Biobehav. Rev.* 126 1–11. 10.1016/j.neubiorev.2021.02.047 33737105 PMC8363507

[B145] Newman P. P. Reza H. (1979). Functional relationships between the hippocampus and the cerebellum: an electrophysiological study of the cat. *J. Physiol.* 287 405–426. 10.1113/jphysiol.1979.sp012667 430426 PMC1281503

[B146] Nicholson A. A. Densmore M. Frewen P. A. Théberge J. Neufeld R. W. McKinnon M. C. (2015). The dissociative subtype of posttraumatic stress disorder: unique resting-state functional connectivity of basolateral and centromedial amygdala complexes. *Neuropsychopharmacology* 40 2317–2326. 10.1038/npp.2015.79 25790021 PMC4538346

[B147] Nishitani N. Nagayasu K. Asaoka N. Yamashiro M. Andoh C. Nagai Y. (2019). Manipulation of dorsal raphe serotonergic neurons modulates active coping to inescapable stress and anxiety-related behaviors in mice and rats. *Neuropsychopharmacology* 44 721–732. 10.1038/s41386-018-0254-y 30377380 PMC6372597

[B148] Nutt D. J. (2008). Relationship of neurotransmitters to the symptoms of major depressive disorder. *J. Clin. Psychiatry* 69 (Suppl E1) 4–7. 18494537

[B149] Onodera S. Hicks T. P. (2009). A comparative neuroanatomical study of the red nucleus of the cat, macaque and human. *PLoS One* 4:e6623. 10.1371/journal.pone.0006623 19675676 PMC2722087

[B150] Palesi F. De Rinaldis A. Castellazzi G. Calamante F. Muhlert N. Chard D. (2017). Contralateral cortico-ponto-cerebellar pathways reconstruction in humans *in vivo*: implications for reciprocal cerebro-cerebellar structural connectivity in motor and non-motor areas. *Sci. Rep.* 7:12841. 10.1038/s41598-017-13079-8 28993670 PMC5634467

[B151] Parker K. L. Narayanan N. S. Andreasen N. C. (2014). The therapeutic potential of the cerebellum in schizophrenia. *Front. Syst. Neurosci.* 8:163. 10.3389/fnsys.2014.00163 25309350 PMC4163988

[B152] Peters G. J. David C. N. Marcus M. D. Smith D. M. (2013). The medial prefrontal cortex is critical for memory retrieval and resolving interference. *Learn. Mem.* 20 201–209. 10.1101/lm.029249.112 23512936 PMC3604648

[B153] Phillips J. R. Hewedi D. H. Eissa A. M. Moustafa A. A. (2015). The cerebellum and psychiatric disorders. *Front. Public Health* 3:66. 10.3389/fpubh.2015.00066 26000269 PMC4419550

[B154] Pierce J. E. Péron J. (2020). The basal ganglia and the cerebellum in human emotion. *Soc. Cogn. Affect. Neurosci.* 15 599–613. 10.1093/scan/nsaa076 32507876 PMC7328022

[B155] Pisano T. J. Dhanerawala Z. M. Kislin M. Bakshinskaya D. Engel E. A. Hansen E. J. (2021). Homologous organization of cerebellar pathways to sensory, motor, and associative forebrain. *Cell Rep.* 36:109721. 10.1016/j.celrep.2021.109721 34551311 PMC8506234

[B156] Poe G. R. Foote S. Eschenko O. Johansen J. P. Bouret S. Aston-Jones G. (2020). Locus coeruleus: a new look at the blue spot. *Nat. Rev. Neurosci.* 21 644–659. 10.1038/s41583-020-0360-9 32943779 PMC8991985

[B157] Pollak Dorocic I. Fürth D. Xuan Y. Johansson Y. Pozzi L. Silberberg G. (2014). A whole-brain atlas of inputs to serotonergic neurons of the dorsal and median raphe nuclei. *Neuron* 83 663–678. 10.1016/j.neuron.2014.07.002 25102561

[B158] Popa L. S. Ebner T. J. (2018). Cerebellum, predictions and errors. *Front. Cell Neurosci.* 12:524. 10.3389/fncel.2018.00524 30697149 PMC6340992

[B159] Prekop H. T. Kroiss A. Rook V. Zagoraiou L. Jessell T. M. Fernandes C. (2018). Sox14 Is required for a specific subset of cerebello-olivary projections. *J. Neurosci.* 38 9539–9550. 10.1523/JNEUROSCI.1456-18.2018 30242051 PMC6706002

[B160] Prentice S. D. Drew T. (2001). Contributions of the reticulospinal system to the postural adjustments occurring during voluntary gait modifications. *J. Neurophysiol.* 85 679–698. 10.1152/jn.2001.85.2.679 11160503

[B161] Proville R. D. Spolidoro M. Guyon N. Dugué G. P. Selimi F. Isope P. (2014). Cerebellum involvement in cortical sensorimotor circuits for the control of voluntary movements. *Nat. Neurosci.* 17 1233–1239. 10.1038/nn.3773 25064850

[B162] Purves D. Augustine G. J. Fitzpatrick D. Hall W. C. LaMantia A.-S. Mooney R. D. (2018). *Neuroscience*, 6th Edn. New York, NY: Oxford University Press, 408–417.

[B163] Ramnani N. (2006). The primate cortico-cerebellar system: anatomy and function. *Nat. Rev. Neurosci.* 7 511–522. 10.1038/nrn1953 16791141

[B164] Ren J. Friedmann D. Xiong J. Liu C. D. Ferguson B. R. Weerakkody T. (2018). Anatomically defined and functionally distinct dorsal raphe serotonin sub-systems. *Cell* 175 472–487 e420. 10.1016/j.cell.2018.07.043 30146164 PMC6173627

[B165] Ren J. Isakova A. Friedmann D. Zeng J. Grutzner S. M. Pun A. (2019). Single-cell transcriptomes and whole-brain projections of serotonin neurons in the mouse dorsal and median raphe nuclei. *Elife* 8:e49424. 10.7554/eLife.49424.043 PMC6812963 31647409

[B166] Ressler K. J. (2010). Amygdala activity, fear, and anxiety: modulation by stress. *Biol. Psychiatry* 67 1117–1119. 10.1016/j.biopsych.2010.04.027 20525501 PMC2882379

[B167] Rochefort C. Lefort J. M. Rondi-Reig L. (2013). The cerebellum: a new key structure in the navigation system. *Front. Neural Circuits* 7:35. 10.3389/fncir.2013.00035 23493515 PMC3595517

[B168] Roelofs K. (2017). Freeze for action: neurobiological mechanisms in animal and human freezing. *Philos. Trans. R. Soc. Lond. B Biol. Sci.* 372:20160206. 10.1098/rstb.2016.0206 28242739 PMC5332864

[B169] Rogers T. D. Dickson P. E. Heck D. H. Goldowitz D. Mittleman G. Blaha C. D. (2011). Connecting the dots of the cerebro-cerebellar role in cognitive function: neuronal pathways for cerebellar modulation of dopamine release in the prefrontal cortex. *Synapse* 65 1204–1212. 10.1002/syn.20960 21638338 PMC3854794

[B170] Rogers T. D. Dickson P. E. McKimm E. Heck D. H. Goldowitz D. Blaha C. D. (2013). Reorganization of circuits underlying cerebellar modulation of prefrontal cortical dopamine in mouse models of autism spectrum disorder. *Cerebellum* 12 547–556. 10.1007/s12311-013-0462-2 23436049 PMC3854915

[B171] Roldán M. Reinoso-Suárez F. (1981). Cerebellar projections to the superior colliculus in the cat. *J. Neurosci.* 1 827–834. 10.1523/JNEUROSCI.01-08-00827.1981 7346586 PMC6564240

[B172] Ross J. A. Van Bockstaele E. J. (2020). The locus coeruleus- norepinephrine system in stress and arousal: unraveling historical, current, and future perspectives. *Front. Psychiatry* 11:601519. 10.3389/fpsyt.2020.601519 33584368 PMC7873441

[B173] Rozeske R. R. Jercog D. Karalis N. Chaudun F. Khoder S. Girard D. (2018). Prefrontal-periaqueductal gray-projecting neurons mediate context fear discrimination. *Neuron* 97 898–910. 10.1016/j.neuron.2017.12.044 29398355

[B174] Rudolph S. Guo C. Pashkovski S. L. Osorno T. Gillis W. F. Krauss J. M. (2020). Cerebellum-specific deletion of the GABAA receptor delta subunit leads to sex-specific disruption of behavior. *Cell Rep.* 33:108338. 10.1016/j.celrep.2020.108338 33147470 PMC7700496

[B175] Ruhé H. G. Mason N. S. Schene A. H. (2007). Mood is indirectly related to serotonin, norepinephrine and dopamine levels in humans: a meta-analysis of monoamine depletion studies. *Mol. Psychiatry* 12 331–359. 10.1038/sj.mp.4001949 17389902

[B176] Ruigrok T. J. H. Teune T. M. (2014). Collateralization of cerebellar output to functionally distinct brainstem areas. A retrograde, non-fluorescent tracing study in the rat. *Front. Syst. Neurosci.* 8:23. 10.3389/fnsys.2014.00023 24600356 PMC3930852

[B177] Russo S. J. Nestler E. J. (2013). The brain reward circuitry in mood disorders. *Nat. Rev. Neurosci.* 14 609–625. 10.1038/nrn3381 23942470 PMC3867253

[B178] Sacchetti B. Baldi E. Lorenzini C. A. Bucherelli C. (2002). Cerebellar role in fear-conditioning consolidation. *Proc. Natl. Acad. Sci. U.S.A.* 99 8406–8411. 10.1073/pnas.112660399 12034877 PMC123080

[B179] Sacchetti B. Scelfo B. Tempia F. Strata P. (2004). Long-term synaptic changes induced in the cerebellar cortex by fear conditioning. *Neuron* 42 973–982. 10.1016/j.neuron.2004.05.012 15207241

[B180] Saleeba C. Dempsey B. Le S. Goodchild A. McMullan S. (2019). A student’s guide to neural circuit tracing. *Front. Neurosci.* 13:897. 10.3389/fnins.2019.00897 31507369 PMC6718611

[B181] Salgado S. Kaplitt M. G. (2015). The nucleus accumbens: a comprehensive review. *Stereotact. Funct. Neurosurg.* 93 75–93. 10.1159/000368279 25720819

[B182] Saper C. B. Loewy A. D. (1980). Efferent connections of the parabrachial nucleus in the rat. *Brain Res.* 197 291–317. 10.1016/0006-8993(80)91117-8 7407557

[B183] Sarno E. Robison A. J. (2018). Emerging role of viral vectors for circuit-specific gene interrogation and manipulation in rodent brain. *Pharmacol. Biochem. Behav.* 174 2–8. 10.1016/j.pbb.2018.04.008 29709585 PMC6369584

[B184] Sathyamurthy A. Barik A. Dobrott C. I. Matson K. J. E. Stoica S. Pursley R. (2020). Cerebellospinal neurons regulate motor performance and motor learning. *Cell Rep.* 31:107595. 10.1016/j.celrep.2020.107595 32402292 PMC7263484

[B185] Schmahmann J. D. Sherman J. C. (1998). The cerebellar cognitive affective syndrome. *Brain* 121(Pt 4) 561–579. 10.1093/brain/121.4.561 9577385

[B186] Schwarz L. A. Miyamichi K. Gao X. J. Beier K. T. Weissbourd B. DeLoach K. E. (2015). Viral-genetic tracing of the input-output organization of a central noradrenaline circuit. *Nature* 524 88–92. 10.1038/nature14600 26131933 PMC4587569

[B187] Sekirnjak C. Vissel B. Bollinger J. Faulstich M. du Lac S. (2003). Purkinje cell synapses target physiologically unique brainstem neurons. *J. Neurosci.* 23 6392–6398. 10.1523/JNEUROSCI.23-15-06392.2003 12867525 PMC6740533

[B188] Sherman S. M. (2016). Thalamus plays a central role in ongoing cortical functioning. *Nat. Neurosci.* 19 533–541. 10.1038/nn.4269 27021938

[B189] Shutoh F. Ohki M. Kitazawa H. Itohara S. Nagao S. (2006). Memory trace of motor learning shifts transsynaptically from cerebellar cortex to nuclei for consolidation. *Neuroscience* 139 767–777. 10.1016/j.neuroscience.2005.12.035 16458438

[B190] Snider R. S. Maiti A. (1976). Cerebellar contributions to the Papez circuit. *J. Neurosci. Res.* 2 133–146. 10.1002/jnr.490020204 950678

[B191] Snider R. S. Maiti A. Snider S. R. (1976). Cerebellar pathways to ventral midbrain and nigra. *Exp. Neurol.* 53 714–728. 10.1016/0014-4886(76)90150-3 1001395

[B192] Sokolov A. A. Miall R. C. Ivry R. B. (2017). The cerebellum: adaptive prediction for movement and cognition. *Trends Cogn. Sci.* 21 313–332. 10.1016/j.tics.2017.02.005 28385461 PMC5477675

[B193] Stanton G. B. (1980). Afferents to oculomotor nuclei from area “Y” in *Macaca mulatta*: an anterograde degeneration study. *J. Comp. Neurol.* 192 377–385. 10.1002/cne.901920211 6772697

[B194] Stapley P. J. Drew T. (2009). The pontomedullary reticular formation contributes to the compensatory postural responses observed following removal of the support surface in the standing cat. *J. Neurophysiol.* 101 1334–1350. 10.1152/jn.91013.2008 19118108

[B195] Steinmetz N. A. Zatka-Haas P. Carandini M. Harris K. D. (2019). Distributed coding of choice, action and engagement across the mouse brain. *Nature* 576 266–273. 10.1038/s41586-019-1787-x 31776518 PMC6913580

[B196] Stoodley C. J. D’Mello A. M. Ellegood J. Jakkamsetti V. Liu P. Nebel M. B. (2017). Altered cerebellar connectivity in autism and cerebellar-mediated rescue of autism-related behaviors in mice. *Nat. Neurosci.* 20 1744–1751. 10.1038/s41593-017-0004-1 29184200 PMC5867894

[B197] Suckow S. K. Deichsel E. L. Ingram S. L. Morgan M. M. Aicher S. A. (2013). Columnar distribution of catecholaminergic neurons in the ventrolateral periaqueductal gray and their relationship to efferent pathways. *Synapse* 67 94–108. 10.1002/syn.21624 23152302 PMC3553663

[B198] Sugihara I. (2011). Compartmentalization of the deep cerebellar nuclei based on afferent projections and aldolase C expression. *Cerebellum* 10 449–463. 10.1007/s12311-010-0226-1 20981512

[B199] Sultan F. Czubayko U. Thier P. (2003). Morphological classification of the rat lateral cerebellar nuclear neurons by principal component analysis. *J. Comp. Neurol.* 455 139–155. 10.1002/cne.10443 12454981

[B200] Supple W. F. Jr. Cranney J. Leaton R. N. (1988). Effects of lesions of the cerebellar vermis on VMH lesion-induced hyperdefensiveness, spontaneous mouse killing, and freezing in rats. *Physiol. Behav.* 42 145–153. 10.1016/0031-9384(88)90290-9 3368533

[B201] Supple W. F. Jr. Leaton R. N. Fanselow M. S. (1987). Effects of cerebellar vermal lesions on species-specific fear responses, neophobia, and taste-aversion learning in rats. *Physiol. Behav.* 39 579–586. 10.1016/0031-9384(87)90156-9 3588702

[B202] Takeuchi T. Duszkiewicz A. J. Sonneborn A. Spooner P. A. Yamasaki M. Watanabe M. (2016). Locus coeruleus and dopaminergic consolidation of everyday memory. *Nature* 537 357–362. 10.1038/nature19325 27602521 PMC5161591

[B203] Tang Z. W. Zhang K. Q. Zhang S. Q. (1987). The fiber projections from the dentate nucleus to the reticular formation of the brain stem in the rabbit. *Anat. Embryol. (Berl)* 175 517–520. 10.1007/BF00309686 3578828

[B204] Tanner M. K. Hake H. S. Bouchet C. A. Greenwood B. N. (2018). Running from fear: exercise modulation of fear extinction. *Neurobiol. Learn. Mem.* 151 28–34. 10.1016/j.nlm.2018.03.021 29614374 PMC6557445

[B205] Taylor N. E. Pei J. Zhang J. Vlasov K. Y. Davis T. Taylor E. (2019). The role of glutamatergic and dopaminergic neurons in the periaqueductal gray/dorsal raphe: separating analgesia and anxiety. *eNeuro* 6 10.1523/ENEURO.0018-18.2019 [Epub ahead of print]. 31058210 PMC6498422

[B206] Tervo D. G. Hwang B. Y. Viswanathan S. Gaj T. Lavzin M. Ritola K. D. (2016). A designer AAV variant permits efficient retrograde access to projection neurons. *Neuron* 92 372–382. 10.1016/j.neuron.2016.09.021 27720486 PMC5872824

[B207] Teune T. M. Burg J. V. D. Moer J. V. D. Voogd J. Ruigrok T. J. (2000). Topography of cerebellar nuclear projections to the brain stem in the rat. *Prog. Brain Res.* 124 141–172. 10.1016/S0079-6123(00)24014-4 10943123

[B208] Thanawalla A. R. Chen A. I. Azim E. (2020). The cerebellar nuclei and dexterous limb movements. *Neuroscience* 450 168–183. 10.1016/j.neuroscience.2020.06.046 32652173 PMC7688491

[B209] Thomas D. M. Kaufman R. P. Sprague J. M. Chambers W. W. (1956). Experimental studies of the vermal cerebellar projections in the brain stem of the cat (fastigiobulbar tract). *J. Anat.* 90 371–385. 13345716 PMC1244800

[B210] Tovote P. Esposito M. S. Botta P. Chaudun F. Fadok J. P. Markovic M. (2016). Midbrain circuits for defensive behaviour. *Nature* 534 206–212. 10.1038/nature17996 27279213

[B211] Tovote P. Fadok J. P. Lüthi A. (2015). Neuronal circuits for fear and anxiety. *Nat. Rev. Neurosci.* 16 317–331. 10.1038/nrn3945 25991441

[B212] Tsutsumi S. Yamazaki M. Miyazaki T. Watanabe M. Sakimura K. Kano M. (2015). Structure-function relationships between aldolase C/zebrin II expression and complex spike synchrony in the cerebellum. *J. Neurosci.* 35 843–852. 10.1523/JNEUROSCI.2170-14.2015 25589776 PMC6605375

[B213] Urban D. J. Zhu H. Marcinkiewcz C. A. Michaelides M. Oshibuchi H. Rhea D. (2016). Elucidation of the behavioral program and neuronal network encoded by dorsal raphe serotonergic neurons. *Neuropsychopharmacology* 41 1404–1415. 10.1038/npp.2015.293 26383016 PMC4793125

[B214] Uusisaari M. Y. Knöpfel T. (2012). Diversity of neuronal elements and circuitry in the cerebellar nuclei. *Cerebellum* 11 420–421. 10.1007/s12311-011-0350-6 22278661

[B215] Vaaga C. E. Brown S. T. Raman I. M. (2020). Cerebellar modulation of synaptic input to freezing-related neurons in the periaqueductal gray. *Elife* 9:e54302. 10.7554/eLife.54302.sa2 PMC7124251 32207681

[B216] Voogd J. (2016). Deiters’ nucleus. its role in cerebellar ideogenesis : the ferdinando rossi memorial lecture. *Cerebellum* 15 54–66. 10.1007/s12311-015-0681-9 26054378 PMC4726724

[B217] Voogd J. Shinoda Y. Ruigrok T. J. H. Sugihara I. (2013). “Cerebellar nuclei and the inferior olivary nuclei: organization and connections,” in *Handbook of the Cerebellum and Cerebellar Disorders*, eds Manto M. Schmahmann J. D. Rossi F. Gruol D. L. Koibuchi N. (Dordrecht: Springer), 377–436. 10.1007/978-94-007-1333-8_19

[B218] Wagner M. J. Luo L. (2020). Neocortex-cerebellum circuits for cognitive processing. *Trends Neurosci.* 43 42–54. 10.1016/j.tins.2019.11.002 31787351 PMC6942222

[B219] Wagner M. J. Savall J. Hernandez O. Mel G. Inan H. Rumyantsev O. (2021). A neural circuit state change underlying skilled movements. *Cell* 184 3731–3747.e3721. 10.1016/j.cell.2021.06.001 34214470 PMC8844704

[B220] Walberg F. (1972). Cerebellovestibular relations: anatomy. *Prog. Brain Res.* 37 361–376. 10.1016/S0079-6123(08)63913-8 4345128

[B221] Walker E. P. Tadi P. (2021). *Neuroanatomy, Nucleus Raphe.* Treasure Island, FL: StatPearls Publishing LLC. 31335079

[B222] Wang D. D. de Hemptinne C. Miocinovic S. Ostrem J. L. Galifianakis N. B. San Luciano M. (2018). Pallidal deep-brain stimulation disrupts pallidal beta oscillations and coherence with primary motor cortex in Parkinson’s Disease. *J. Neurosci.* 38 4556–4568. 10.1523/JNEUROSCI.0431-18.2018 29661966 PMC5943981

[B223] Wang X. Yu S. Y. Ren Z. De Zeeuw C. I. Gao Z. (2020). A FN-MdV pathway and its role in cerebellar multimodular control of sensorimotor behavior. *Nat. Commun.* 11:6050. 10.1038/s41467-020-19960-x 33247191 PMC7695696

[B224] Watabe-Uchida M. Zhu L. Ogawa S. K. Vamanrao A. Uchida N. (2012). Whole-brain mapping of direct inputs to midbrain dopamine neurons. *Neuron* 74 858–873. 10.1016/j.neuron.2012.03.017 22681690

[B225] Watson G. D. R. Hughes R. N. Petter E. A. Fallon I. P. Kim N. Severino F. P. U. (2021). Thalamic projections to the subthalamic nucleus contribute to movement initiation and rescue of parkinsonian symptoms. *Sci. Adv.* 7:eabe9192. 10.1126/sciadv.abe9192 33547085 PMC7864574

[B226] Watson T. C. Becker N. Apps R. Jones M. W. (2014). Back to front: cerebellar connections and interactions with the prefrontal cortex. *Front. Syst. Neurosci.* 8:4. 10.3389/fnsys.2014.00004 24550789 PMC3912388

[B227] Watson T. C. Obiang P. Torres-Herraez A. Watilliaux A. Coulon P. Rochefort C. (2019). Anatomical and physiological foundations of cerebello-hippocampal interaction. *Elife* 8:e41896. 10.7554/eLife.41896.027 PMC6579515 31205000

[B228] Wolff M. Vann S. D. (2019). The cognitive thalamus as a gateway to mental representations. *J. Neurosci.* 39 3–14. 10.1523/JNEUROSCI.0479-18.2018 30389839 PMC6325267

[B229] Xiao L. Bornmann C. Hatstatt-Burklé L. Scheiffele P. (2018). Regulation of striatal cells and goal-directed behavior by cerebellar outputs. *Nat. Commun.* 9:3133. 10.1038/s41467-018-05565-y 30087345 PMC6081479

[B230] Xu P. Chen A. Li Y. Xing X. Lu H. (2019). Medial prefrontal cortex in neurological diseases. *Physiol. Genomics* 51 432–442. 10.1152/physiolgenomics.00006.2019 31373533 PMC6766703

[B231] Xu X. Holmes T. C. Luo M. H. Beier K. T. Horwitz G. D. Zhao F. (2020). Viral vectors for neural circuit mapping and recent advances in trans-synaptic anterograde tracers. *Neuron* 107 1029–1047. 10.1016/j.neuron.2020.07.010 32755550 PMC7530073

[B232] You I. J. Wright S. R. Garcia-Garcia A. L. Tapper A. R. Gardner P. D. Koob G. F. (2016). 5-HT1A autoreceptors in the dorsal raphe nucleus convey vulnerability to compulsive cocaine seeking. *Neuropsychopharmacology* 41 1210–1222. 10.1038/npp.2015.268 26324408 PMC4793105

[B233] Yu W. Krook-Magnuson E. (2015). Cognitive collaborations: bidirectional functional connectivity between the cerebellum and the hippocampus. *Front. Syst. Neurosci.* 9:177. 10.3389/fnsys.2015.00177 26732845 PMC4686701

[B234] Zahm D. S. Cheng A. Y. Lee T. J. Ghobadi C. W. Schwartz Z. M. Geisler S. (2011). Inputs to the midbrain dopaminergic complex in the rat, with emphasis on extended amygdala-recipient sectors. *J. Comp. Neurol.* 519 3159–3188. 10.1002/cne.22670 21618227 PMC3174784

[B235] Zeidler Z. Hoffmann K. Krook-Magnuson E. (2020). HippoBellum: acute cerebellar modulation alters hippocampal dynamics and function. *J. Neurosci.* 40 6910–6926. 10.1523/JNEUROSCI.0763-20.2020 32769107 PMC7470923

[B236] Zhou J. Brown A. M. Lackey E. P. Arancillo M. Lin T. Sillitoe R. V. (2020). Purkinje cell neurotransmission patterns cerebellar basket cells into zonal modules defined by distinct pinceau sizes. *Elife* 9:e55569. 10.7554/eLife.55569.sa2 PMC7561353 32990595

[B237] Zhou M. Melin M. D. Xu W. Südhof T. C. (2020). Dysfunction of parvalbumin neurons in the cerebellar nuclei produces an action tremor. *J. Clin. Invest.* 130 5142–5156. 10.1172/JCI135802 32634124 PMC7524475

[B238] Zingg B. Chou X. L. Zhang Z. G. Mesik L. Liang F. Tao H. W. (2017). AAV-mediated anterograde transsynaptic tagging: mapping corticocollicular input-defined neural pathways for defense behaviors. *Neuron* 93 33–47. 10.1016/j.neuron.2016.11.045 27989459 PMC5538794

